# ELOVL2 mediated stabilization of AR contributes to enzalutamide resistance in prostate cancer

**DOI:** 10.3389/fcell.2025.1598400

**Published:** 2025-06-09

**Authors:** Jinpeng Cen, Jiading Guo, Xianzi Zeng, Xianlu Song, Shengdong Ge, Mingkun Chen, Qianyi Li, Yuzhong Yu, Daojun Lv, Shanchao Zhao

**Affiliations:** ^1^ Department of Urology, Nanfang Hospital, Southern Medical University, Guangzhou, Guangdong, China; ^2^ Department of Urology, Guangzhou Institute of Cancer Research, the Affiliated Cancer Hospital, Guangzhou Medical University, Guangzhou, Guangdong, China; ^3^ Department of Urology, The Fifth Affiliated Hospital, Southern Medical University, Guangzhou, Guangdong, China; ^4^ Department of Radiotherapy, Guangzhou Institute of Cancer Research, The Affiliated Cancer Hospital, Guangzhou Medical University, Guangzhou, Guangdong, China; ^5^ Department of Urology, The Fourth Affiliated Hospital, Guangzhou Medical University, Guangzhou, Guangdong, China; ^6^ First Clinical Medical College, Southern Medical University, Guangzhou, Guangdong, China; ^7^ Department of Urology, Guangdong Provincial Key Laboratory of Major Obstetric Diseases, Guangdong Provincial Clinical Research Center for Obstetrics and Gynecology, The Third Affiliated Hospital, Guangzhou Medical University, Guangzhou, Guangdong, China

**Keywords:** ELOVL2, enzalutamide resistance, androgen receptor, prostate cancer, CRPC

## Abstract

**Introduction:**

To investigate the molecular mechanisms underlying enzalutamide resistance in castration-resistant prostate cancer (CRPC) and explore potential therapeutic strategies to overcome resistance.

**Methods:**

We conducted comprehensive bioinformatic analysis using LNCaP/enzalutamide-resistant cells to identify key pathways associated with resistance. Functional validation was performed through targeted inhibition of the elongation of very-long chain fatty acid protein 2 (ELOVL2), followed by assays to assess cancer cell proliferation and enzalutamide sensitivity. Mechanistic studies were conducted to evaluate the impact of ELOVL2 on the ubiquitin-proteasome system and AR signaling pathways.

**Results:**

Bioinformatic analysis revealed that activation of fatty acid metabolism, particularly through upregulation of ELOVL2, plays a critical role in driving enzalutamide resistance in PCa. Functional studies demonstrated that targeted inhibition of ELOVL2 significantly suppressed cancer cell proliferation and restored enzalutamide sensitivity in resistant cells. Mechanistically, ELOVL2 facilitates enzalutamide resistance by impairing the ubiquitin-proteasome system, leading to the subsequent activation of AR signaling pathways.

**Discussion:**

Our findings demonstrate that ELOVL2 drives enzalutamide resistance in CRPC by stabilizing AR through inhibition of ubiquitin-proteasome-mediated degradation. Targeting ELOVL2 represents a promising therapeutic strategy to overcome resistance in CRPC, with potential to improve clinical outcomes for patients.

## 1 Introduction

The androgen receptor (AR) serves as a critical driver in the pathogenesis and progression of prostate cancer (PCa) (Siegel et al., 2025). Androgen deprivation therapy (ADT) represents the standard therapeutic intervention for patients presenting with biochemical recurrence after radical prostatectomy. Nevertheless, prolonged ADT frequently induces treatment resistance, culminating in the development of castration-resistant prostate cancer (CRPC) (Wang et al., 2021). While the emergence of next-generation AR inhibitors, particularly enzalutamide (Enz), has significantly advanced the therapeutic landscape, clinical outcomes remain suboptimal. This is primarily due to the inevitable development of enzalutamide resistance in most patients, which severely limits therapeutic options and correlates with poor prognosis (Mckay et al., 2021). Consequently, the identification of novel therapeutic targets to overcome enzalutamide resistance has become a paramount priority in PCa research.

The reactivation of AR signaling has been widely recognized as a predominant mechanism underlying enzalutamide resistance, playing a pivotal role in PCa progression, particularly in the advancement of CRPC. Extensive research has elucidated that AR reactivation can be attributed to multiple molecular alterations, including AR gene amplification, mutations within the AR ligand-binding domain (LBD), expression of AR splice variants, and activation of alternative signaling pathways that functionally interact with AR signaling in an androgen-deprived microenvironment (Chan et al., 2015). Furthermore, emerging evidence indicates that enzaluta. Recent studies have revealed an additional layer of complexity, demonstrating that enzalutamide treatment can paradoxically enhance AR protein stability through post-translational modifications, including phosphorylation and ubiquitination alterations (De las pozas et al., 2018; Rodriguez tirado et al., 2024; Choudhury et al., 2024; Wang et al., 2025),despite its intended function as an AR signaling inhibitor (Li et al., 2022). This stabilization effect contributes to persistent AR signaling even in the presence of the drug. mide treatment itself may induce selective outgrowth of AR-low dependent clones, drive phenotypic plasticity of tumor cells, and activate distinct oncogenic pathways that facilitate disease progression (Shiota et al., 2024; Li et al., 2024; Su et al., 2022). Crucially, quantitative proteomic analyses demonstrate that resistant cell lines exhibit longer AR half-lives compared to treatment-naïve cells, directly correlating with reduced proteasomal degradation efficiency (Astapova et al., 2021). These findings underscore the critical importance of elucidating the molecular mechanisms governing AR protein stabilization and maintenance during enzalutamide treatment, as understanding these processes may reveal novel therapeutic vulnerabilities that could be exploited to prevent or reverse treatment resistance. Such insights may provide crucial foundations for developing novel strategies to overcome therapeutic resistance in PCa, particularly through combination therapies targeting both AR synthesis and degradation pathways.

ELOVL2, a key member of the elongation of very-long-chain fatty acids (ELOVL) enzyme family, encodes a transmembrane protein that catalyzes the elongation of various long-chain fatty acids, including saturated, monounsaturated, and polyunsaturated fatty acids (Jakobsson et al., 2006). Beyond its fundamental enzymatic function, emerging evidence reveals that dysregulation of ELOVL2 expression exhibits context-dependent and often paradoxical roles in tumorigenesis across different cancer types. In renal cell carcinoma, elevated ELOVL2 expression correlates with poor prognosis and promotes tumor progression through anti-apoptotic mechanisms (Tanaka et al., 2022). In contrast, ELOVL2 overexpression exerts tumor-suppressive effects in neuroblastoma by inhibiting cellular proliferation (Ding et al., 2019). Particularly in PCa, ELOVL2 demonstrates a unique tumor-suppressive function, where its high expression is associated with favorable clinical outcomes, while its knockdown enhances malignant phenotypes including proliferation, migration, and invasion in PCa cells (Hu et al., 2022). However, whether this tumor-suppressive role persists in advanced PCa under therapeutic pressure (enzalutamide treatment) remains unexplored. Given the known metabolic reprogramming in castration-resistant progression and the context-dependent roles of ELOVL2 across cancers (oncogenic in renal carcinoma vs. tumor-suppressive in neuroblastoma), we hypothesized that ELOVL2 may exhibit stage-specific functional plasticity in PCa. Despite these significant findings, current research has predominantly focused on elucidating the role of ELOVL2 in PCa pathogenesis, with a notable paucity of systematic investigations exploring its potential involvement in therapeutic resistance. Intriguingly, preliminary studies have suggested that ELOVL2 deficiency may function as a tumor suppressor in certain contexts, particularly in attenuating tamoxifen resistance through distinct molecular mechanisms (Kim et al., 2023). Moreover, recent work (Liu et al., 2023; Zhu et al., 2016; Lu et al., 2023) has implicated fatty acid metabolism enzymes in modulating protein stability of key oncogenic drivers, suggesting a potential link between ELOVL2 activity and AR protein turnover. Building upon these observations, the present study seeks to comprehensively investigate the functional role and underlying mechanisms of ELOVL2 in enzalutamide resistance in PCa, which aiming to provide novel insights into overcoming therapeutic resistance in advanced PCa.

In this study, we implemented an integrated research strategy combining computational analysis of multiple GEO datasets from LNCaP cells with experimental validation to identify key epigenetically regulated genes involved in enzalutamide resistance development. Our systematic approach revealed ELOVL2 as a crucial molecular determinant in the acquisition resistance of enzalutamide. To further characterize its functional role, we conducted extensive investigations to evaluate the therapeutic efficacy of ELOVL2 suppression in re-sensitizing resistant PCa cells to enzalutamide treatment. Through detailed mechanistic exploration, we discovered that ELOVL2 inhibition reverses therapeutic resistance predominantly by regulating the ubiquitin-proteasome pathway, thereby establishing a novel molecular link between fatty acid metabolism and AR signaling in advanced PCa. These findings provide significant insights into the molecular basis of treatment resistance and identify ELOVL2 as a potential therapeutic target for overcoming enzalutamide resistance in CRPC patients.

## 2 Materials and methods

### 2.1 Bioinformatic analysis

To validate our findings, we analyzed four publicly available RNA sequencing datasets from the Gene Expression Omnibus (GEO) database: GSE163539 (LNCaP cells treated with DHT), GSE128749 (LNCaP cells treated with R1881), GSE150807 (LNCaP cells treated with enzalutamide), and GSE163240 (enzalutamide-resistant LNCaP cells). Differential expression analysis of ELOVL2 was performed across all datasets using standardized bioinformatics pipelines.

### 2.2 Patients and samples

For clinical validation of our findings, we collected a cohort of nine prostate tissue specimens from patients with localized PCa who underwent either prostate biopsy or radical prostatectomy at the Fifth Affiliated Hospital of Southern Medical University. Among these specimens, two cases were histologically confirmed as enzalutamide-resistant PCa. Comprehensive clinical and pathological characteristics of these patients are detailed in Supplementary Table S1. All participants provided written informed consent prior to sample collection, and the study protocol was approved by the Institutional Review Board of the Fifth Affiliated Hospital of Southern Medical University (Approval No. 2025-MNWK-K-001), ensuring compliance with international ethical standards for human subject research.

### 2.3 Immunohistochemistry (IHC)

IHC analysis was performed following our previously established protocol (Lv et al., 2019). Briefly, paraffin-embedded tissue blocks were sectioned at 10 μm thickness and mounted on slides. After deparaffinization and rehydration through a graded ethanol series, endogenous peroxidase activity was quenched by incubating sections in 0.3% hydrogen peroxide for 15 min at room temperature. Tissue sections were then incubated with primary rabbit anti-ELOVL2 antibody (1:400 dilution; 20308-1-AP,proteintech) overnight at 4°C. Following primary antibody incubation, slides were treated with horseradish peroxidase-conjugated anti-rabbit secondary antibodies (Dako, K4003) for 1 h at room temperature. Immunoreactivity was visualized using liquid 3,3′-diaminobenzidine tetrahydrochloride (DAB) substrate (Dako, K3468), followed by counterstaining with Mayer’s hematoxylin. Whole slide images were acquired using a panoramic MIDI scanner (3Dhistech, Budapest, Hungary) at ×20 magnification. Quantitative analysis of immunohistochemical staining was performed using ImageJ software (NIH, Bethesda, MD, United States of America) to determine the percentage of DAB-positive areas relative to total tissue area.

### 2.4 Cell culture and generation of enzalutamide-resistant PCa cells

The human benign prostatic hyperplastic cell line BPH-1 and PCa cell lines (PC-3, DU145, 22Rv1, C4-2, and LNCaP) were obtained from the Stem Cell Bank of the Chinese Academy of Sciences. All cell lines were cultured in RPMI-1640 medium supplemented with 10% fetal bovine serum (FBS; Hyclone), 25 U/mL penicillin, and 25 μg/mL streptomycin. Cells were maintained at 37°C in a humidified atmosphere containing 5% CO_2_.

To establish enzalutamide-resistant PCa cell lines, LNCaP and C4-2 cells were first adapted to androgen-deprived conditions by culturing in phenol red-free RPMI-1640 medium supplemented with 10% charcoal-stripped FBS for 1 week. Enzalutamide resistance was induced by stepwise exposure to increasing concentrations of enzalutamide (5 μM, 10 μM, 20 μM, and finally 30 µM), with each concentration maintained for 7 days. Following the establishment of resistance, the cells were maintained in medium containing 10 µM enzalutamide. The resulting enzalutamide-resistant cell lines, designated as LNCaP-enzR and C4-2-enzR, were used for subsequent experiments.

### 2.5 Construction of ELOVL2-knockdown cell lines

To investigate the functional role of ELOVL2, gene silencing was achieved using both transient siRNA transfection and stable lentiviral transduction approaches. For transient knockdown, Lipofectamine 3,000 (Invitrogen, Carlsbad, CA, United States of America,L3000008) was used to transfect cells with ELOVL2-specific small interfering RNAs (siRNAs) or negative control (NC) siRNA, following the manufacturer’s protocol. Three independent siRNAs targeting ELOVL2, along with their corresponding sequences (provided in Supplementary Table S2), were designed and synthesized by RiboBio Co. (Guangzhou, China).

For stable ELOVL2 knockdown, lentiviral particles containing short hairpin RNA (shRNA) targeting ELOVL2 were generated using the pLKO.1 vector system (GeneChem Bio-Medical Biotechnology, Shanghai, China). Lentiviral transduction was performed according to the manufacturer’s instructions, followed by puromycin selection (2 μg/mL) to establish stable shELOVL2 cell lines. Knockdown efficiency was validated at both mRNA and protein levels using quantitative real-time PCR and Western blot analysis, respectively.

### 2.6 RNA extraction and quantitative real-time PCR (qRT-PCR) assays

Total RNA was extracted from PCa cells using the Total RNA Extraction Kit (Seven, China), following the manufacturer’s instructions. cDNA synthesis was performed using the PrimeScript RT Reagent Kit (TaKaRa, Japan,RR047A), and qRT-PCR was carried out using the TB-Green PCR Master Mix Kit (TaKaRa, Japan,RR430B). Glyceraldehyde 3-phosphate dehydrogenase (GAPDH) was used as an internal control, and relative gene expression levels were calculated using the 2^−ΔΔCt^ method. Primer sequences for target genes are provided in Supplementary Table S3.

### 2.7 Western blotting

PCa cells were lysed using RIPA buffer supplemented with Proteinase inhibitors, protein phosphatase inhibitors, PMSF. Protein concentrations were quantified using the BCA assay, and equal amounts of protein (30 µg) were separated by 10% SDS-PAGE and transferred onto polyvinylidene fluoride (PVDF) membranes.

Membranes were blocked with 5% skimmed milk in TBST for 1 h at room temperature and then incubated overnight at 4°C with the following primary antibodies: anti-Tubulin (Cell Signaling Technology, United States of America, 2128S), anti-ELOVL2 (Abcam, United States of America,ab176327), anti-AR (Cell Signaling Technology, United States of America), and anti-Ubiquitin Enzymes (Abcam, United States of America,ab134953). After washing, membranes were incubated with horseradish peroxidase (HRP)-conjugated secondary antibodies for 1 h at room temperature. Protein bands were visualized using an enhanced chemiluminescence (ECL) kit (Pierce Biotechnology, United States of America, 32109) and quantified using ImageJ software.

### 2.8 Cell viability and enzalutamide sensitivity assay

Cell viability was assessed using the Cell Counting Kit-8 (CCK-8) assay. Briefly, PCa cells were seeded at a density of 2,000 cells per well in 96-well plates and allowed to adhere overnight. After treatment, 10% CCK-8 reagent was added to each well, and plates were incubated at 37°C for 2 h. Absorbance was measured at 450 nm using a microplate reader.

For enzalutamide sensitivity assays, 1 × 10^4^ cells were seeded per well in 96-well plates and transfected with siRNA or plasmids as indicated. After 24 h, cells were treated with enzalutamide at final concentrations of 0, 0.05, 0.1, 0.5, and 2 µM (with a final ethanol concentration of 0.1%). Following 24 h of treatment, 10 µL of CCK-8 solution was added to each well, and plates were incubated for 90 min. Absorbance was measured at 450 nm, and the half-maximal inhibitory concentration (IC_50_) was calculated.

### 2.9 Colony formation assay

PCa cells were seeded at a density of 300 cells per well in 6-well plates and cultured for 2 weeks to allow colony formation. Colonies were fixed with 4% paraformaldehyde for 15 min and stained with 0.5% Giemsa solution for 30 min. Colonies containing >50 cells were counted under a microscope. Each experimental condition was performed in triplicate, and the assay was repeated three times independently.

### 2.10 Protein degradation half-life assay

To assess the degradation rate of AR protein, PCa cells were treated with cycloheximide (CHX, 10 μg/mL, HY-12320), a protein synthesis inhibitor (Astapova et al., 2021). Cells in good growth condition were divided into two groups: control and ELOVL2 knockdown. Each group was seeded in 6-well plates at 50% confluence, with three replicate wells per group. When cells reached 70%–80% confluence, CHX was added to block new protein synthesis. Cells were harvested at 0, 4, and 8 h post-treatment, and total protein was extracted. AR protein levels were analyzed by Western blot, and band intensities were quantified using ImageJ software. The half-life (t_1_/_2_) of AR protein was calculated based on the degradation kinetics.

### 2.11 Ubiquitination assay

To determine whether the ubiquitin-proteasome system mediates AR protein degradation, co-immunoprecipitation (Co-IP) was performed using protein extracts from PCa cells. Cells were cultured to a density equivalent to one 15 cm^2^ dish or three 10 cm^2^ dishes, washed with PBS, trypsinized, and collected. After resuspension and centrifugation to remove residual medium and trypsin, cells were lysed in ice-cold lysis buffer supplemented with 1% protease inhibitor, phosphatase inhibitor, and PMSF. The lysate was incubated on ice for 30 min and centrifuged to collect the supernatant. The supernatant was divided into two groups: Input (100 μL) and IP (400 μL). The Input group was used to determine protein concentration, mixed with loading buffer, and stored at −80°C for subsequent analysis. The IP group was incubated with AR primary antibody (2–5 μg) at 4°C overnight. Protein A/G beads (40–50 μL) were then added to the IP group and incubated at 4°C overnight. Beads were washed 2–3 times with PBST:PMSF (100:1) and PBS:PMSF (100:1) to remove non-specific binding. Bound proteins were eluted using 1× loading buffer (diluted with wash buffer) and heat-denatured at 95°C for 5 min before storage at −80°C. Finally, AR protein and its ubiquitinated forms were detected by Western blot. The ubiquitination levels of AR were quantified to assess the role of the ubiquitin-proteasome pathway in AR degradation.

### 2.12 MG132 proteasome inhibition assay

To investigate the involvement of the ubiquitin-proteasome system in AR protein degradation, ELOVL2-knockdown enzalutamide-resistant PCa cells and control cells were treated with MG132 (MCE, HY-13259), a proteasome inhibitor. Cells were seeded in 6-well plates at 70% confluence and cultured until reaching 90% confluence. The experimental group was treated with complete medium containing 10 μg/mL CHX and 10 μM MG132, while the control group was treated with complete medium containing 10 μg/mL CHX alone. Cells were incubated at 37°C for 8 h to allow proteasome inhibition and protein degradation analysis. After treatment, cells were washed with PBS, harvested, and lysed in RIPA buffer supplemented with 1% PMSF, protease inhibitors, and phosphatase inhibitors. The lysates were incubated on ice for 30 min and centrifuged at 12,000 rpm, 4°C for 30 min. The supernatants were collected and mixed with 25% volume of 4× loading buffer, followed by storage at −80°C for subsequent analysis. Target protein expression, particularly AR, was detected by Western blot. Band intensities were quantified using ImageJ software, and statistical analysis was performed to assess the effects of MG132 on AR protein stability in ELOVL2-knockdown and control cells.

### 2.13 Statistical analysis

Data are presented as mean ± standard error of the mean (SEM) unless otherwise stated. Statistical significance was determined using unpaired two-tailed Student’s t-tests or one-way ANOVA, as appropriate. Significance levels were defined as  *p < 0.05,  **p < 0.01,  ***p < 0.001, and  ****p < 0.0001. All statistical analyses were performed using SPSS software (version 22.0; IBM Corporation, Armonk, NY, United States of America).

## 3 Results

### 3.1 Identification of ELOVL2 overexpression in enzalutamide-resistant PCa cells

**FIGURE 1:**
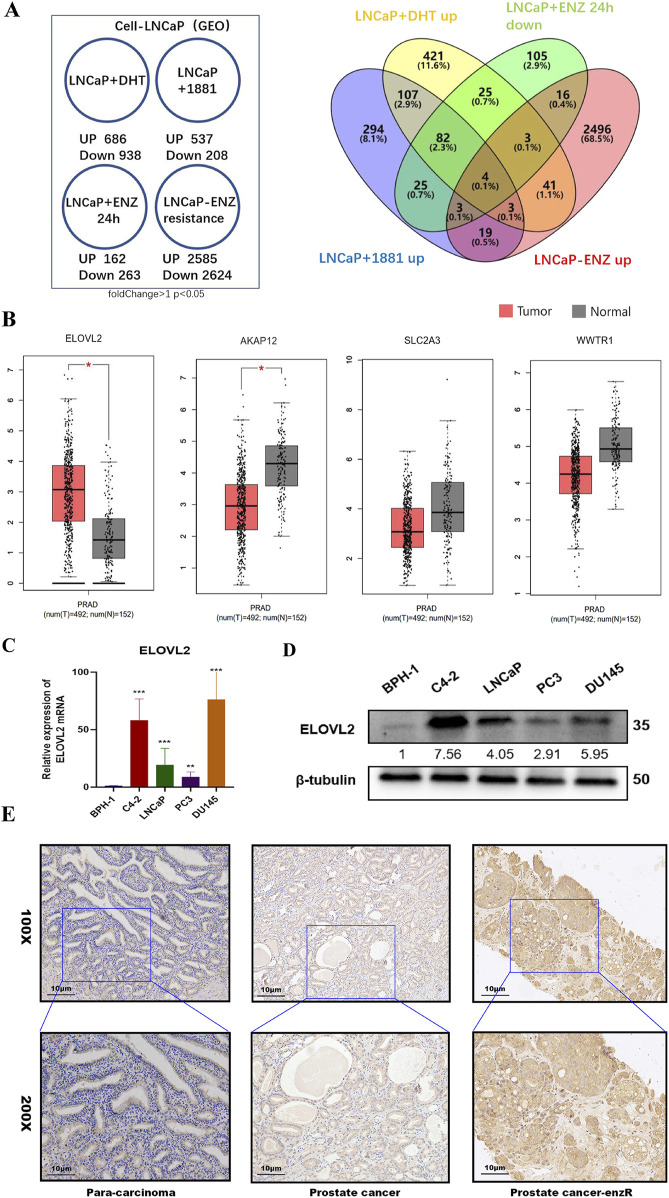
Identification of ELOVL2 as a Key Regulator in Enzalutamide Resistance. **(A)** Bioinformatics analysis of GEO datasets was performed to identify differentially expressed genes associated with enzalutamide resistance in PCa. **(B)** Comparative analysis of ELOVL2 expression levels between prostate tumor tissues and adjacent normal tissues, demonstrating significant upregulation in malignant samples. **(C)** qRT-PCR analysis revealing differential expression of ELOVL2 in benign prostatic hyperplasia (BPH) tissues versus PCa cell lines (LNCaP, C4-2, PC3, and DU145),Data are presented as mean ± SD;  **P < 0.01.  ***P < 0.001. ****P < 0.0001. **(D)** Western blot analysis revealing protein expression level of ELOVL2 in benign prostatic hyperplasia (BPH) tissues versus PCa cell lines (LNCaP, C4-2, PC3, and DU145). **(E)** Representative immunohistochemical images showing elevated ELOVL2 expression (brown-yellow staining) in PCa tissues compared to non-tumorous tissues.

To identify genes potentially involved in enzalutamide resistance, we conducted a comprehensive analysis of multiple enzalutamide-resistant PCa models. Four independent transcriptomic datasets were analyzed to compare gene expression profiles between enzalutamide-resistant (ENZ-R) and enzalutamide-sensitive samples. Through cross-comparison of differentially expressed genes, we identified a consensus signature of four genes (AKAP12, ELOVL2, SLC2A3, and WWTR1) that were significantly upregulated in ENZ-R samples (>1.5-fold change, P < 0.05; Figure 1A).

To validate these findings, we quantified the mRNA levels of these genes in the TCGA dataset and a panel of PCa cell lines (LNCaP, C4-2, PC3, and DU145). ELOVL2 exhibited the most pronounced upregulation in both PCa tissues (Figure 1B) and PCa cell lines compared to the benign prostatic hyperplasia (BPH) cell line BPH-1(Figure 1C; Supplementary Figure S1). Western blot analysis further confirmed elevated ELOVL2 protein expression in three PCa cell lines (C4-2, LNCaP, and DU145) relative to BPH-1 cells(Figure 1D). IHC analysis of archived paraffin-embedded PCa specimens (n = 9) revealed significantly higher ELOVL2 expression in tumor tissues, particularly in enzalutamide-resistant cases, whereas adjacent non-tumorous tissues showed minimal expression (Supplementary Figure S2).

These multi-level analyses demonstrate that ELOVL2 is consistently overexpressed in enzalutamide-resistant PCa and suggest its potential role as a critical mediator of therapeutic resistance. These findings highlight ELOVL2 as a promising biomarker and therapeutic target for overcoming enzalutamide resistance in advanced PCa.

### 3.2 ELOVL2 is upregulated in enzalutamide-resistant PCa cells and positively correlates with AR expression

**FIGURE 2:**
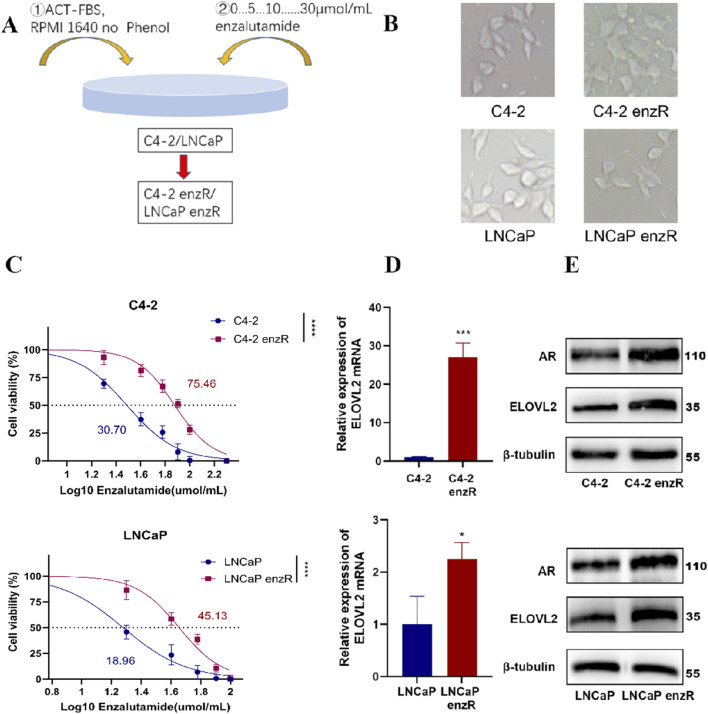
Characterization of Enzalutamide-Resistant PCa Cell Lines. **(A)** Schematic illustration of the experimental workflow for generating AR-positive enzalutamide-resistant PCa cell lines (LNCaP-enzR and C4-2-enzR). **(B)** Representative images showing morphological changes in LNCaP and C4-2 cells following the development of enzalutamide resistance. LNCaP-enzR cells exhibited a shrunken phenotype with elongated synapses, while C4-2-enzR cells transitioned from a spindle-shaped to a polygonal morphology with dendritic extensions. **(C)** Dose-response curves and half-maximal inhibitory concentration (IC_50_) values for enzalutamide in parental and resistant cell lines. IC_50_ values were significantly higher in resistant cells (C4-2-enzR: 75.46 µM, 95% CI: 72.02–78.99,  ****P < 0.0001; LNCaP-enzR: 45.13 µM, 95% CI: 41.45–48.75,  ****P < 0.01) compared to their parental counterparts (C4-2: 30.70 µM, 95% CI: 28.31–33.12; LNCaP: 18.96 µM, 95% CI: 15.85–21.61). **(D)** qRT-PCR analysis demonstrating upregulated expression of ELOVL2 in LNCaP-enzR and C4-2-enzR cells compared to their parental cell lines, Data are presented as mean ± SD,  *P < 0.05,  ***P < 0.001. **(E)** Western blot analysis demonstrating upregulated expression of ELOVL2 and AR in LNCaP-enzR and C4-2-enzR cells compared to their parental cell lines.

LNCaP and C4-2 cell lines are widely used to model the biological behavior of primary tumor cells from CRPC patients and tumor cells following ADT, given their clinical relevance and origins (Guo et al., 2024; Mourkioti et al., 2023). To investigate the mechanisms of enzalutamide resistance, we established enzalutamide-resistant sublines derived from LNCaP and C4-2 cells, designated as LNCaP-enzR and C4-2-enzR, respectively (Figure 2A).

Notably, both LNCaP-enzR and C4-2-enzR cells exhibited distinct morphological changes compared to their parental counterparts (Figure 2B). LNCaP-enzR cells displayed a slightly shrunken phenotype with elongated synaptic extensions, while C4-2-enzR cells transitioned from a spindle-shaped morphology to a polygonal form with prominent dendrite-like structures. Functional characterization confirmed increased enzalutamide resistance in these sublines, as evidenced by a significantly higher half-maximal inhibitory concentration (IC_50_) compared to parental cells (Figure 2C). Furthermore, to assess the reversibility of the ENZ-R phenotype, we cultured ENZ-R cells in enzalutamide-free medium for 7 days and re-exposed them to the drug. No significant cell death was observed upon re-challenge, and repeated IC_50_ measurements remained consistent with the initial resistance profile (Figure 2C; Supplementary Figure S3). These data indicate that the ENZ-R phenotype is stable even after short-term drug withdrawal, underscoring the robustness of our model system.

Furthermore, qPCR analysis demonstrated significant upregulation of ELOVL2 mRNA in both LNCaP-enzR and C4-2-enzR cells compared to their parental counterparts (Figure 2D). This transcriptional induction was consistently reflected at the protein level, as Western blot analysis revealed markedly elevated expression of both ELOVL2 and AR in the resistant cells (Figure 2E; Supplementary Figure S4). The concordant upregulation of ELOVL2 at both mRNA and protein levels, along with increased AR expression, suggests that prolonged enzalutamide treatment induces coordinated upregulation of ELOVL2 and AR in resistant cells. Importantly, the positive correlation between ELOVL2 and AR levels in enzalutamide-resistant PCa cells reveals a potential functional interplay between ELOVL2-mediated fatty acid metabolism and AR signaling in driving therapeutic resistance.

### 3.3 ELOVL2 inhibition suppresses the growth of enzalutamide-resistant PCa cells

**FIGURE 3:**
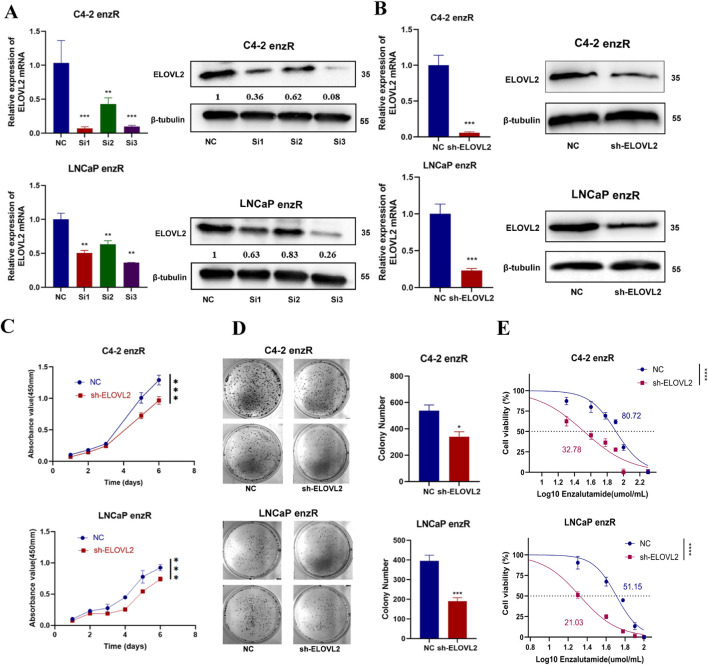
ELOVL2 inhibition suppresses growth and restores enzalutamide sensitivity in resistant PCa cells. **(A)** Validation of ELOVL2 knockdown efficiency using three independent small interfering RNAs (siRNAs) by qRT-PCR and Western blot analysis. siRNA#3 showed the most potent knockdown effect and was selected for subsequent experiments. **(B)** Stable ELOVL2 knockdown using lentiviral shRNA (derived from siRNA#3 sequence). Knockdown efficiency was confirmed by qRT-PCR and Western blot. **(C)** Cell proliferation assessed by CCK-8 assay in C4-2-enzR and LNCaP-enzR cells following ELOVL2 knockdown. Data represent mean ± SD (n = 5), ***p < 0.001. **(D)** Colony formation assay demonstrating the proliferative capacity of C4-2-enzR and LNCaP-enzR cells after ELOVL2 inhibition. Colonies were counted after 14 days (mean ± SD, n = 3), *p < 0.05, ***p < 0.001. **(E)** Dose-response curves and calculated half-maximal inhibitory concentration (IC_50_) values for enzalutamide in ELOVL2-depleted cells. ELOVL2 knockdown significantly reduced IC_50_ values in both C4-2-enzR (32.78 μM, 95% CI: 27.95–37.54; ***p < 0.0001) and LNCaP-enzR (21.03 μM, 95% CI: 19.62–22.36; ***p < 0.0001) cells compared to control (C4-2-NC: 80.72 μM, 95% CI: 75.26–86.48; LNCaP-NC: 51.15 μM, 95% CI: 47.92–54.33).

To further investigate the functional role of ELOVL2 in prostate cancer (PCa), we employed siRNA-mediated knockdown to deplete ELOVL2 expression in enzalutamide-resistant LNCaP-enzR and C4-2-enzR cells. Among the three siRNAs tested, SI3 exhibited the most significant knockdown efficiency, as confirmed by qRT-PCR and Western blotting at the mRNA and protein levels, respectively (Figure 3A). Subsequently, we generated stable shELOVL2 cell lines using lentiviral delivery of SI3. Further validation by qRT-PCR and Western blotting demonstrated a marked reduction in ELOVL2 expression in both cell lines (Figure 3B; Supplementary Figure S5).

Functional assays revealed that ELOVL2 knockdown markedly inhibited cell proliferation, as evidenced by CCK-8 assays and colony formation experiments (Figures 3C,D; Supplementary Figure S6). To determine whether ELOVL2 contributes to enzalutamide resistance, we assessed the sensitivity of ELOVL2-depleted cells to enzalutamide. Notably, ELOVL2 silencing significantly reduced IC_50_ of enzalutamide in both LNCaP-enzR (IC_50_: 21.03 µM vs. 51.15 µM,  ****<0.0001) and C4-2-enzR (IC_50_: 32.78 µM vs. 80.72 µM,  ****<0.0001) cells (Figure 3E). Furthermore, we silenced ELOVL2 in both parental cell lines (LNCaP and C4-2) and measured their IC_50_ values. The results showed no significant differences in IC_50_ changes upon ELOVL2 silencing in these cells (Supplementary Figure S7), further highlighting the specific role of ELOVL2 in mediating enzalutamide resistance within the CRPC context.

These findings collectively demonstrate that ELOVL2 promotes the growth of enzalutamide-resistant PCa cells and plays a critical role in maintaining therapeutic resistance. Targeting ELOVL2 may therefore represent a promising strategy to overcome enzalutamide resistance in advanced PCa.

### 3.4 ELOVL2 promotes AR protein stabilization by inhibiting the ubiquitin-proteasome system

**FIGURE 4:**
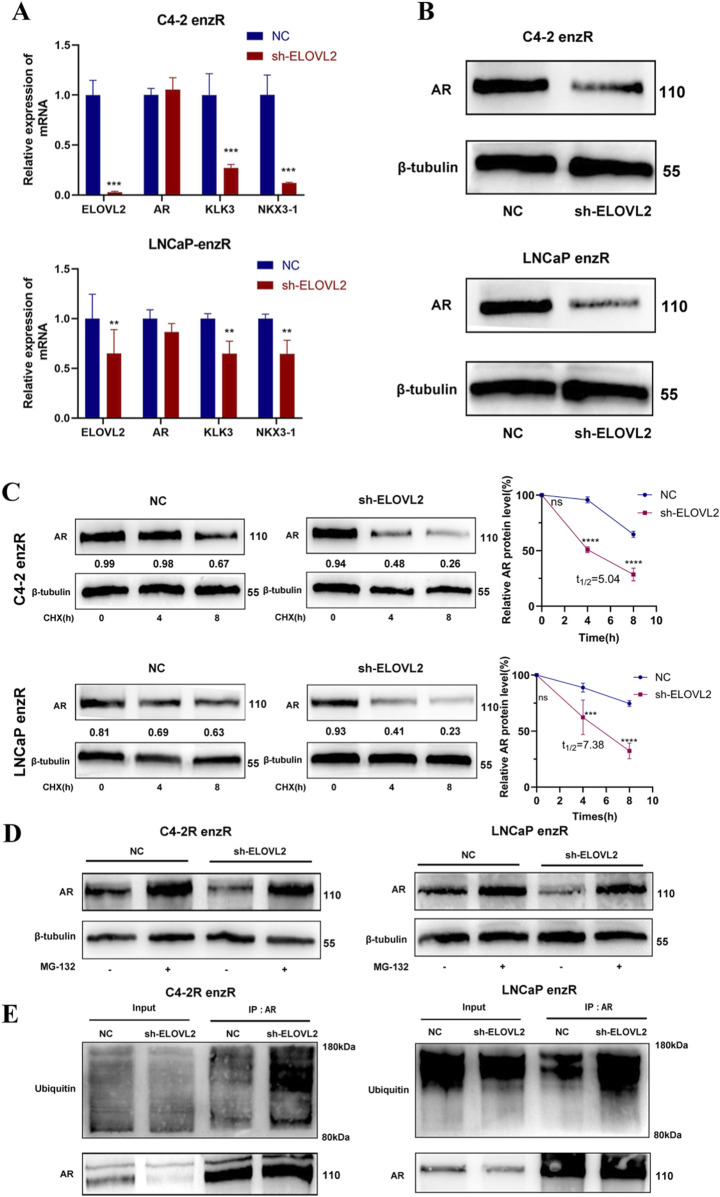
ELOVL2 Regulates AR Protein Stability via the Ubiquitin-Proteasome Pathway. **(A)** qRT-PCR analysis of AR mRNA and its downstream target genes (Klk3 and NKX3-1) in ELOVL2-depleted PCa cell lines. ELOVL2 knockdown did not significantly alter AR mRNA levels but reduced the expression of Klk3 and NKX3-1. **(B)** Western blot analysis showing decreased AR protein levels in ELOVL2-knockdown C4-2-enzR and LNCaP-enzR cells compared to controls. **(C)** AR protein stability assay using cycloheximide treatment. AR protein degradation was significantly accelerated in ELOVL2-depleted cells, C4-2-enzR-shELOVL2 t_1/2_ = 5.04 h, LNCaP-enzR-shELOVL2 t_1/2_ = 7.38 h,ns no significant difference, ***P < 0.001, ****P < 0.0001). **(D)** Western blot analysis of AR protein levels in enzR cells treated with or without the proteasome inhibitor MG132 (10 μM, 8 h). “-” and “+” indicate untreated control and MG132 treatment, respectively. **(E)** CO-IP and Western blot analysis demonstrating increased ubiquitination of AR protein in ELOVL2-knockdown enzR cells, indicating enhanced proteasomal degradation.

AR is the primary target of enzalutamide, and AR-associated mechanisms are closely linked to therapeutic resistance in PCa. To investigate whether ELOVL2 influences AR signaling, we examined the expression of AR and its downstream coactivators (Klk3 and NKX3-1) in enzalutamide-resistant PCa cells. While ELOVL2 knockdown did not significantly alter AR mRNA levels, it markedly reduced the expression of Klk3 and NKX3-1, which are downstream targets of AR signaling (Figure 4A). Western blot analysis further revealed that ELOVL2 depletion led to a significant decrease in AR protein levels in enzalutamide-resistant cells (Figure 4B; Supplementary Figure S8), suggesting that ELOVL2 regulates AR at the post-translational level.

To explore this mechanism, we treated ELOVL2-depleted cells with cycloheximide, a protein synthesis inhibitor, and observed accelerated AR protein degradation compared to control cells (Figure 4C). This finding indicates that ELOVL2 plays a critical role in maintaining AR protein stability. To further elucidate the pathway involved, we treated ELOVL2-knockdown C4-2-enzR and LNCaP-enzR cells with the proteasome inhibitor MG132. Western blot analysis demonstrated that MG132 treatment significantly attenuated AR protein degradation, confirming the involvement of the ubiquitin-proteasome system (Figure 4D).

To directly assess the role of ELOVL2 in AR ubiquitination, we performed co-immunoprecipitation assays. Results showed that ELOVL2 knockdown significantly increased the ubiquitination levels of AR protein (Figure 4E), providing direct evidence that ELOVL2 regulates AR stability through the ubiquitin-proteasome pathway.

Collectively, these findings demonstrate that ELOVL2 maybe upregulates AR protein expression by inhibiting the ubiquitin-proteasome pathway, thereby enhancing AR signaling and contributing to enzalutamide resistance in PCa cells.

## 4 Discussion

The androgen-AR signaling pathway is a well-established driver of PCa progression, particularly in AR-positive disease. As a result, ADT has emerged as the cornerstone treatment for advanced PCa (Shelan et al., 2024). However, despite initial therapeutic responses, patients often experience disease relapse and tumor progression, leading to the development of CRPC. Clinical studies have demonstrated that CRPC remains dependent on AR signaling, as evidenced by the therapeutic efficacy of second-generation AR pathway inhibitors, such as enzalutamide (Li et al., 2024). Nevertheless, the clinical benefits of enzalutamide are often short-lived, with the majority of patients rapidly developing resistance. This underscores the urgent need to elucidate the molecular mechanisms underlying enzalutamide resistance in CRPC and to identify novel therapeutic targets and strategies to overcome this challenge. These efforts have become a major focus of PCa research, with the ultimate goal of improving outcomes for patients with advanced disease.

In this study, we identified ELOVL2 as a key mediator of enzalutamide resistance in PCa. Functional experiments demonstrated that ELOVL2 depletion significantly inhibits the growth of enzalutamide-resistant PCa cells, highlighting its critical role in therapeutic resistance. ELOVL2, a member of the fatty acid elongation enzyme family, catalyzes the elongation of very long-chain polyunsaturated fatty acids (VLC-PUFAs), including the conversion of docosapentaenoic acid (DPA, 22:5n-3) to docosahexaenoic acid (DHA, 22:6n-3) (Gregory et al., 2013). This enzymatic activity has drawn increasing attention to the role of lipid metabolism and metabolomics in cancer biology. The function of ELOVL2 in cancer appears to be context-dependent, with studies reporting both tumor-suppressive and oncogenic roles. In breast cancer, ELOVL2 acts as a tumor suppressor by attenuating tamoxifen resistance (Jeong et al., 2021), whereas in renal cell carcinoma, it promotes tumor progression by inhibiting apoptosis. This duality suggests that ELOVL2’s role is highly influenced by the tumor microenvironment and cancer type. Notably, a 2022 study revealed that ELOVL2 is significantly upregulated in PCa compared to normal prostate tissue and regulates cancer cell behavior through INPP4B signaling, further underscoring its importance in PCa progression (Hu et al., 2022). Our findings align with these observations, demonstrating that ELOVL2 is highly expressed in PCa and plays a complex role in drug resistance. Specifically, we show that ELOVL2 contributes to enzalutamide resistance but can also restore drug sensitivity when targeted, suggesting that its biological functions may vary across different stages of PCa progression. These results highlight the potential of ELOVL2 as a therapeutic target for overcoming enzalutamide resistance in advanced PCa.

It was wildly known that the AR is tightly regulated in normal prostate tissue, where it is predominantly expressed in luminal epithelial cells to maintain prostate growth and development. Dysregulation of AR expression is a hallmark of PCa pathogenesis and progression, as AR plays a central role in regulating key cellular processes, including proliferation, migration, invasion, and differentiation (Lamb et al., 2014). In this study, we demonstrated that ELOVL2 modulates AR protein stability at the post-translational level, leading to increased AR protein levels in PCa cells and contributing to enzalutamide resistance. Post-transcriptional or translational regulation of AR has emerged as a critical mechanism in PCa. For instance, METTL3-mediated m6A methylation has been shown to regulate AR alternative splicing, highlighting the role of RNA modifications in AR signaling (Haigh et al., 2022). Notably, m6A levels are significantly upregulated in CRPC compared to hormone-sensitive PCa, suggesting a potential link between RNA modification and therapeutic resistance (Li et al., 2023). Our findings further reveal that ELOVL2 regulates AR degradation through the ubiquitin-proteasome system, as demonstrated by MG132 treatment experiments. This aligns with previous studies showing that AR stability is modulated by ubiquitin-proteasome pathways. For example, deltex proteins, a family of E3 ubiquitin ligases, have been shown to form oligomeric complexes that recognize and regulate ADP-ribosylated AR (Vela-rodríguez et al., 2024). Additionally, SMYD2-mediated methylation and phosphorylation of AR influence its ubiquitination and proteasomal degradation, thereby altering the AR transcriptome in CRPC cells (Li et al., 2024). These collective findings underscore the complexity of AR regulation in PCa and highlight the ubiquitin-proteasome system as a critical node for therapeutic intervention. Our study adds to this growing body of evidence by identifying ELOVL2 as a novel regulator of AR stability and enzalutamide resistance, providing new insights into the molecular mechanisms underlying CRPC progression.

Despite the significant findings of this study, several challenges and unanswered questions remain. As a fatty acid elongase, the precise mechanism by which ELOVL2 regulates the ubiquitin-proteasome pathway is still unclear and warrants further investigation. Additionally, it remains to be determined whether ELOVL2 can influence the biological activity or expression levels of AR mRNA, which could provide deeper insights into its role in AR signaling. Interestingly, other members of the fatty acid elongase family have also been implicated in cancer progression. For instance, ELOVL5, another enzyme in this family, has been shown to alter mitochondrial morphology and function, leading to increased reactive oxygen species production and subsequent suppression of PCa cell proliferation, tumor growth, and metastasis (Centenera et al., 2021). Similarly, ELOVL6 and ELOVL7 have been reported to be overexpressed in various tumors and contribute to cancer progression (Marien et al., 2016; Tolkach et al., 2015; Feng et al., 2016). However, whether these elongases can regulate the ubiquitin-proteasome system, as observed with ELOVL2, remains unexplored. These gaps in knowledge highlight the need for further research to elucidate the broader role of the ELOVL family in cancer biology, particularly their potential interactions with protein degradation pathways. Understanding these mechanisms could uncover novel therapeutic targets and strategies for overcoming resistance in advanced PCa and other malignancies.

Overall, the identification of ELOVL2 as a key regulator of AR signaling represents a significant breakthrough in understanding enzalutamide resistance in advanced PCa. This discovery not only elucidates a novel molecular mechanism underlying therapeutic resistance but also opens a promising new avenue for the development of targeted therapies. By inhibiting ELOVL2, it may be possible to restore enzalutamide sensitivity and improve outcomes for patients with advanced PCa. These findings underscore the potential of ELOVL2 as a therapeutic target and highlight the importance of further research to translate these insights into clinical applications.

## 5 Conclusion

**FIGURE 5:**
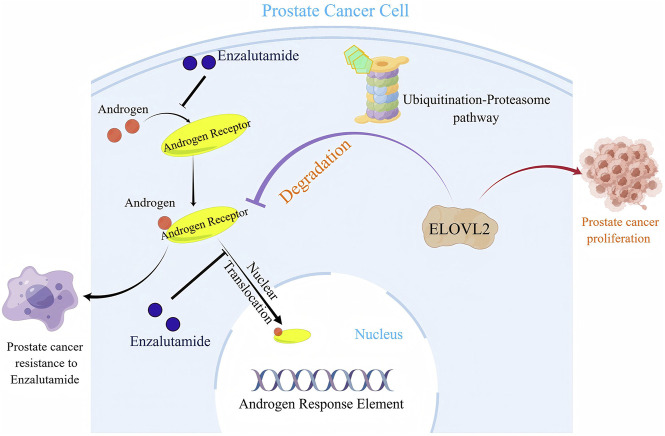
A schematic illustration of the molecular mechanism by which ELOVL2-mediated stabilization of AR contributes to Enzalutamide resistance in PCa.

In summary, our study identifies ELOVL2 as a critical regulator of AR signaling that drives enzalutamide resistance in PCa. Mechanistically, ELOVL2 stabilizes AR protein by inhibiting the ubiquitin-proteasome pathway, thereby sustaining AR signaling and promoting therapeutic resistance (Figure 5). These findings highlight the potential of targeting ELOVL2 as a novel therapeutic strategy to overcome enzalutamide resistance in advanced PCa, offering a promising avenue for improving clinical outcomes in patients with CRPC.

## Data Availability

The datasets presented in this study can be found in online repositories. The names of the repository/repositories and accession number(s) can be found below: https://www.ncbi.nlm.nih.gov/geo/, GSE163539; https://www.ncbi.nlm.nih.gov/geo/, GSE128749; https://www.ncbi.nlm.nih.gov/geo/, GSE150807; https://www.ncbi.nlm.nih.gov/geo/, GSE163240.
