# Correction: ELOVL2 mediated stabilization of AR contributes to enzalutamide resistance in prostate cancer

**DOI:** 10.3389/fcell.2025.1646699

**Published:** 2025-06-25

**Authors:** Jinpeng Cen, Jiading Guo, Xianzi Zeng, Xianlu Song, Shengdong Ge, Mingkun Chen, Qianyi Li, Yuzhong Yu, Daojun Lv, Shanchao Zhao

**Affiliations:** ^1^ Department of Urology, Nanfang Hospital, Southern Medical University, Guangzhou, Guangdong, China; ^2^ Department of Urology, Guangzhou Institute of Cancer Research, the Affiliated Cancer Hospital, Guangzhou Medical University, Guangzhou, Guangdong, China; ^3^ Department of Urology, The Fifth Affiliated Hospital, Southern Medical University, Guangzhou, Guangdong, China; ^4^ Department of Radiotherapy, Guangzhou Institute of Cancer Research, The Affiliated Cancer Hospital, Guangzhou Medical University, Guangzhou, Guangdong, China; ^5^ Department of Urology, The Fourth Affiliated Hospital, Guangzhou Medical University, Guangzhou, Guangdong, China; ^6^ First Clinical Medical College, Southern Medical University, Guangzhou, Guangdong, China; ^7^ Department of Urology, Guangdong Provincial Key Laboratory of Major Obstetric Diseases, Guangdong Provincial Clinical Research Center for Obstetrics and Gynecology, The Third Affiliated Hospital, Guangzhou Medical University, Guangzhou, Guangdong, China

**Keywords:** ELOVL2, enzalutamide resistance, androgen receptor, prostate cancer, CRPC

In the published article, there was an error in the legend for Figure 3E. Due to an oversight, the IC50 value for LNCaP-NC was incorrectly reported as:

**FIGURE 3:**
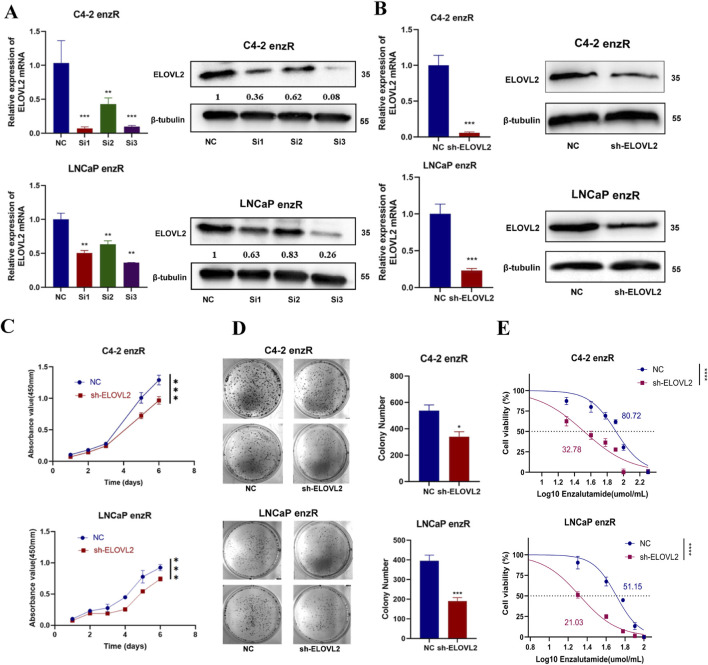
ELOVL2 inhibition suppresses growth and restores enzalutamide sensitivity in resistant PCa cells. **(A)**Validation of ELOVL2 knockdown efficiency using three independent small interfering RNAs (siRNAs) by qRT-PCR and Western blot analysis. siRNA#3 showed the most potent knockdown effect and was selected for subsequent experiments. **(B)** Stable ELOVL2 knockdown using lentiviral shRNA (derived from siRNA#3 sequence). Knockdown efficiency was confirmed by qRT-PCR and Western blot. **(C)** Cell proliferation assessed by CCK-8 assay in C4-2-enzR and LNCaP-enzR cells following ELOVL2 knockdown. Data represent mean ± SD (n = 5), ***p < 0.001. **(D)** Colony formation assay demonstrating the proliferative capacity of C4-2-enzR and LNCaP-enzR cells after ELOVL2 inhibition. Colonies were counted after 14 days (mean ± SD, n = 3), *p < 0.05, ***p < 0.001. **(E)** Dose-response curves and calculated half-maximal inhibitory concentration (IC_50_) values for enzalutamide in ELOVL2-depleted cells. ELOVL2 knockdown significantly reduced IC_50_ values in both C4-2-enzR (32.78 µM, 95% CI: 27.95–37.54; ****p < 0.0001) and LNCaP-enzR (21.03 µM, 95% CI: 19.62–22.36; ****p < 0.0001) cells compared to control (C4-2-NC: 80.72 µM, 95% CI: 75.26–86.48; LNCaP-NC: 51.15 µM, 95% CI: 47.92–54.33).

“LNCaP-NC: 21.03 µM, 95% CI: 19.62–22.36.”

The corrected legend appears below.

“LNCaP-NC: 51.15 µM, 95% CI: 47.92–54.33.”

The authors apologize for this error and confirm that it does not affect the scientific conclusions of the article. The original article has been updated accordingly.

In the published article, there was an error in Figure 3E as presented. The visualization results for Figure 3E were correctly submitted and approved during the first round of revisions. However, due to an oversight on our part, this figure was inadvertently omitted in the second revised version. The corrected Figure 3E and its accompanying caption—Figure 3 ELOVL2 inhibition suppresses growth and restores enzalutamide sensitivity in resistant PCa cells—are provided below.

The authors apologize for this error and state that this does not change the scientific conclusions of the article in any way. The original article has been updated.

