# Supplementary figures and images for: ELOVL2 mediated stabilization of AR contributes to enzalutamide resistance in prostate cancer
Source: Front Cell Dev Biol. 2025 Jun 9;13:1598400. doi: 10.3389/fcell.2025.1598400 (PMC12183063; doi:10.3389/fcell.2025.1598400)

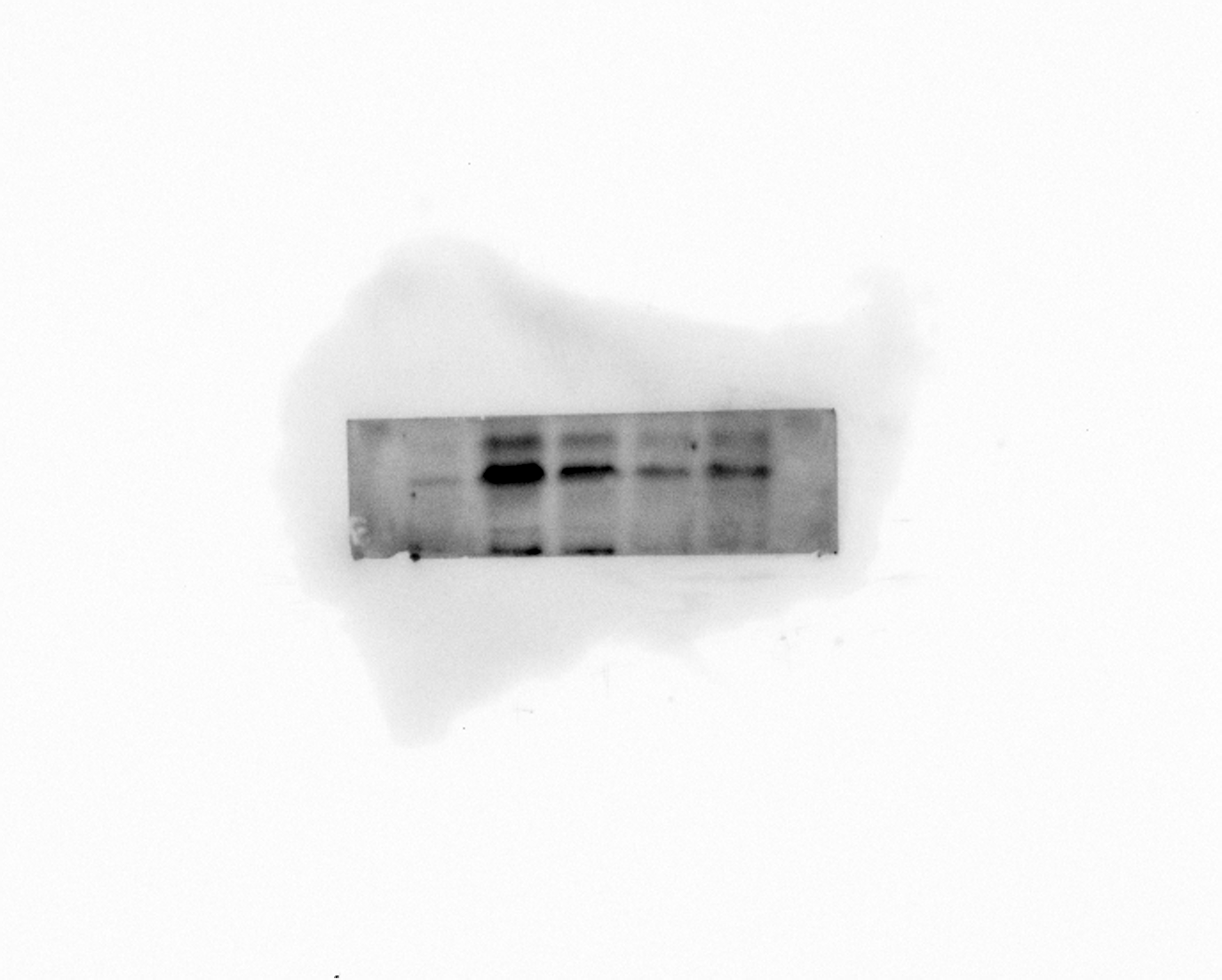

Supplement: Supplementary file 1 [file Supplementaryfile1.zip › Figure-Western blot images(revision-2025.05.22)/Figure 1. Western blot images/Figure1D. ELOVL2.Tif]

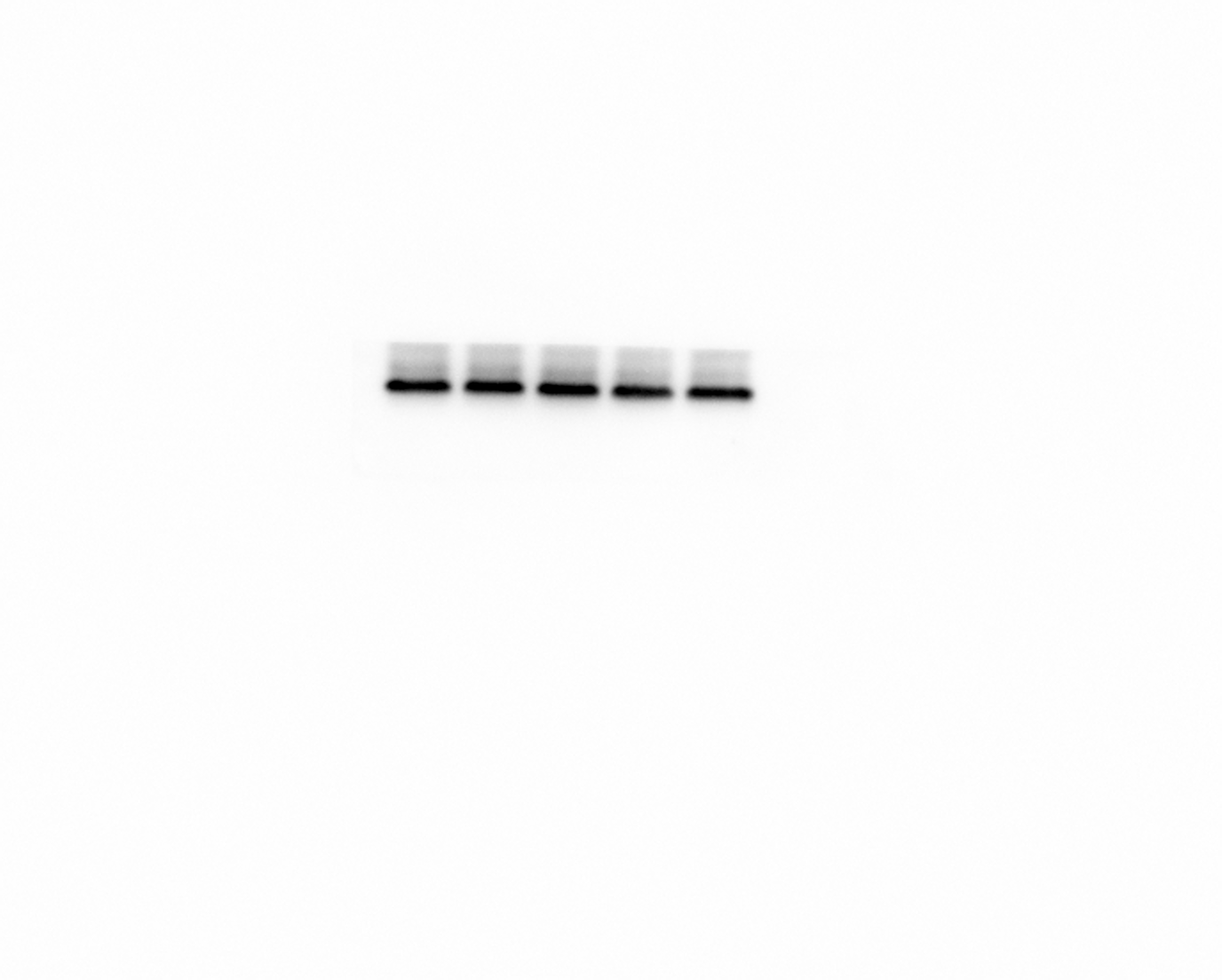

Supplement: Supplementary file 1 [file Supplementaryfile1.zip › Figure-Western blot images(revision-2025.05.22)/Figure 1. Western blot images/Figure1D.β-tubulin.Tif]

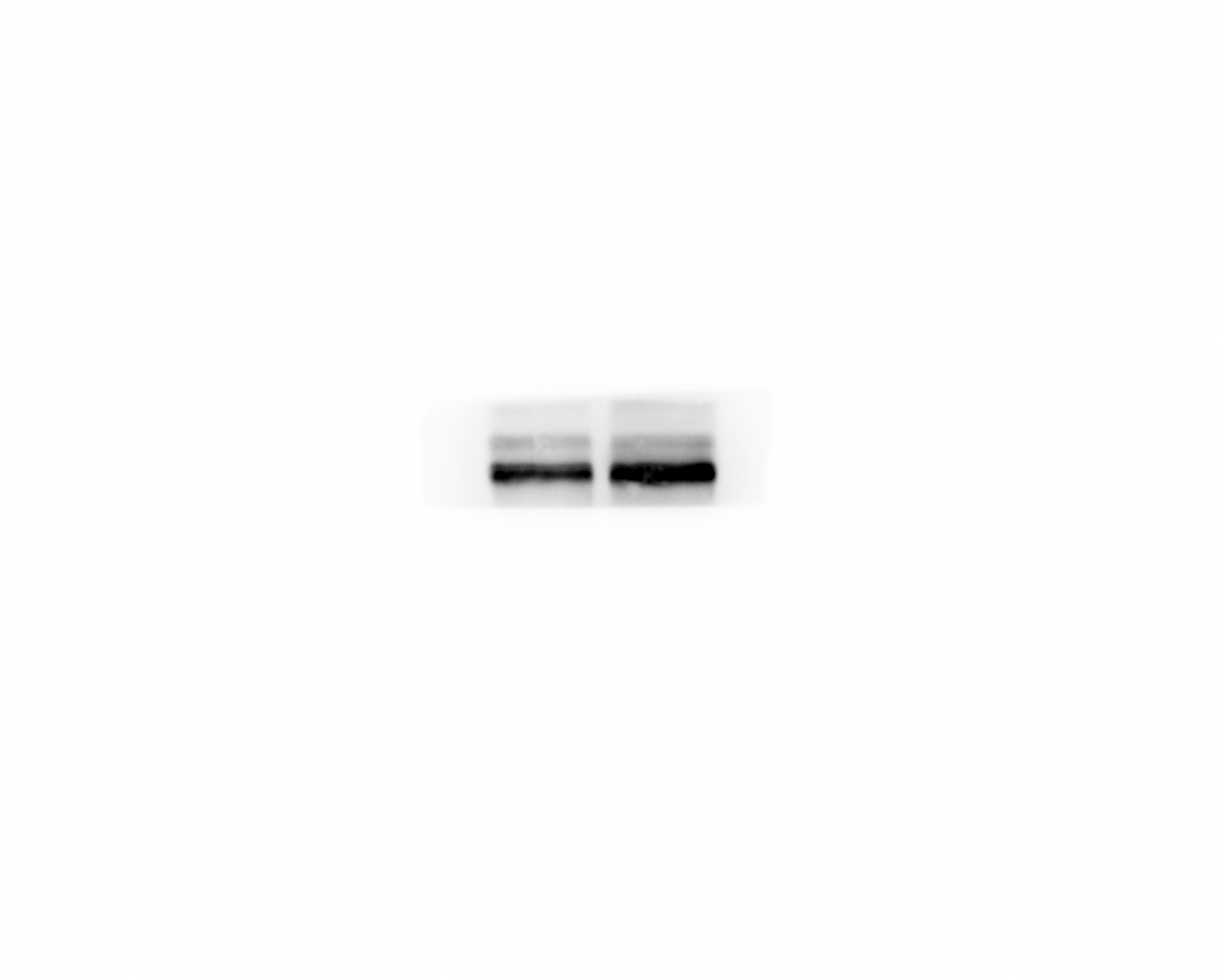

Supplement: Supplementary file 1 [file Supplementaryfile1.zip › Figure-Western blot images(revision-2025.05.22)/Figure 2. Western blot images/Figure2D.C4-2.AR.Tif]

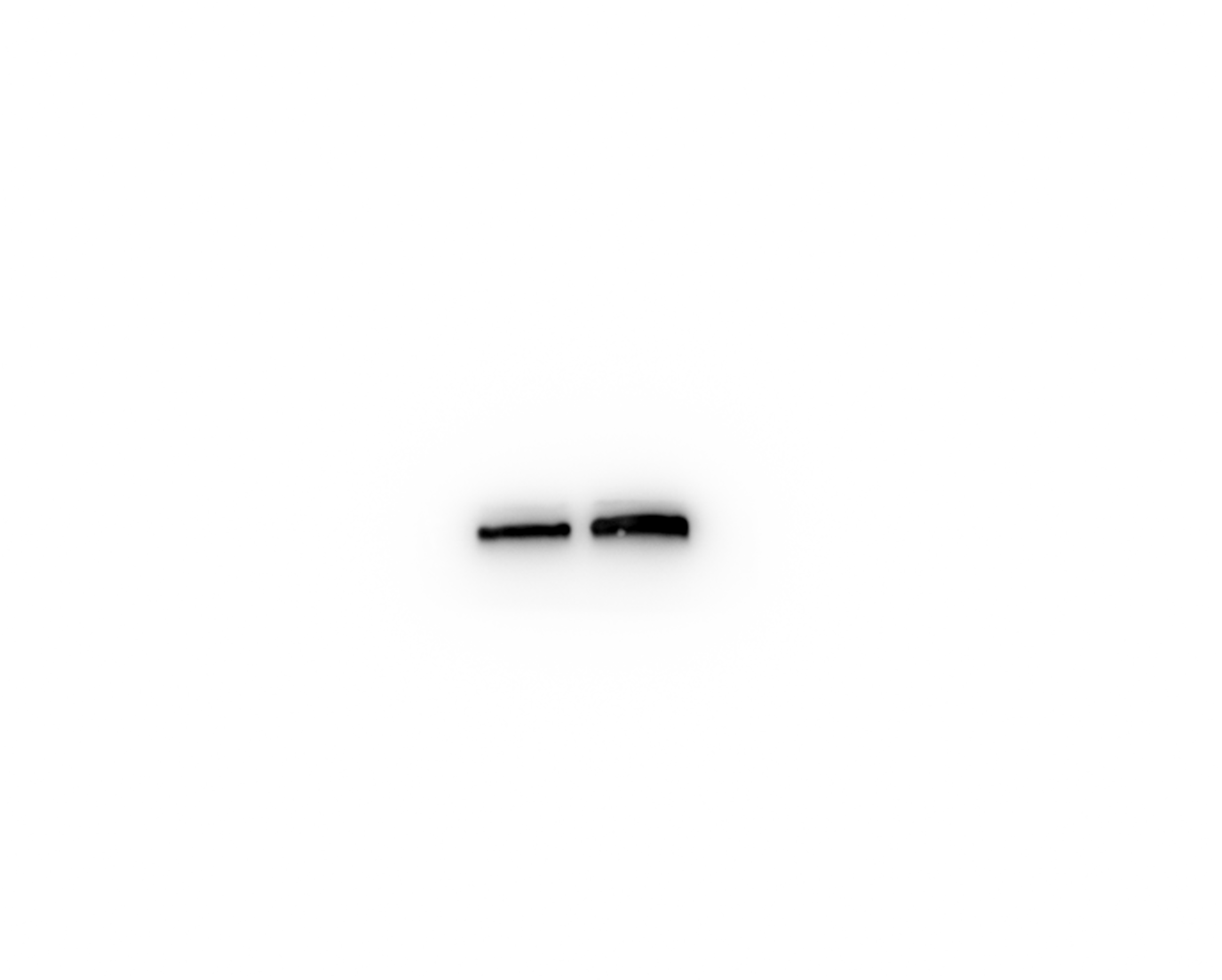

Supplement: Supplementary file 1 [file Supplementaryfile1.zip › Figure-Western blot images(revision-2025.05.22)/Figure 2. Western blot images/Figure2D.C4-2.ELOVL2.Tif]

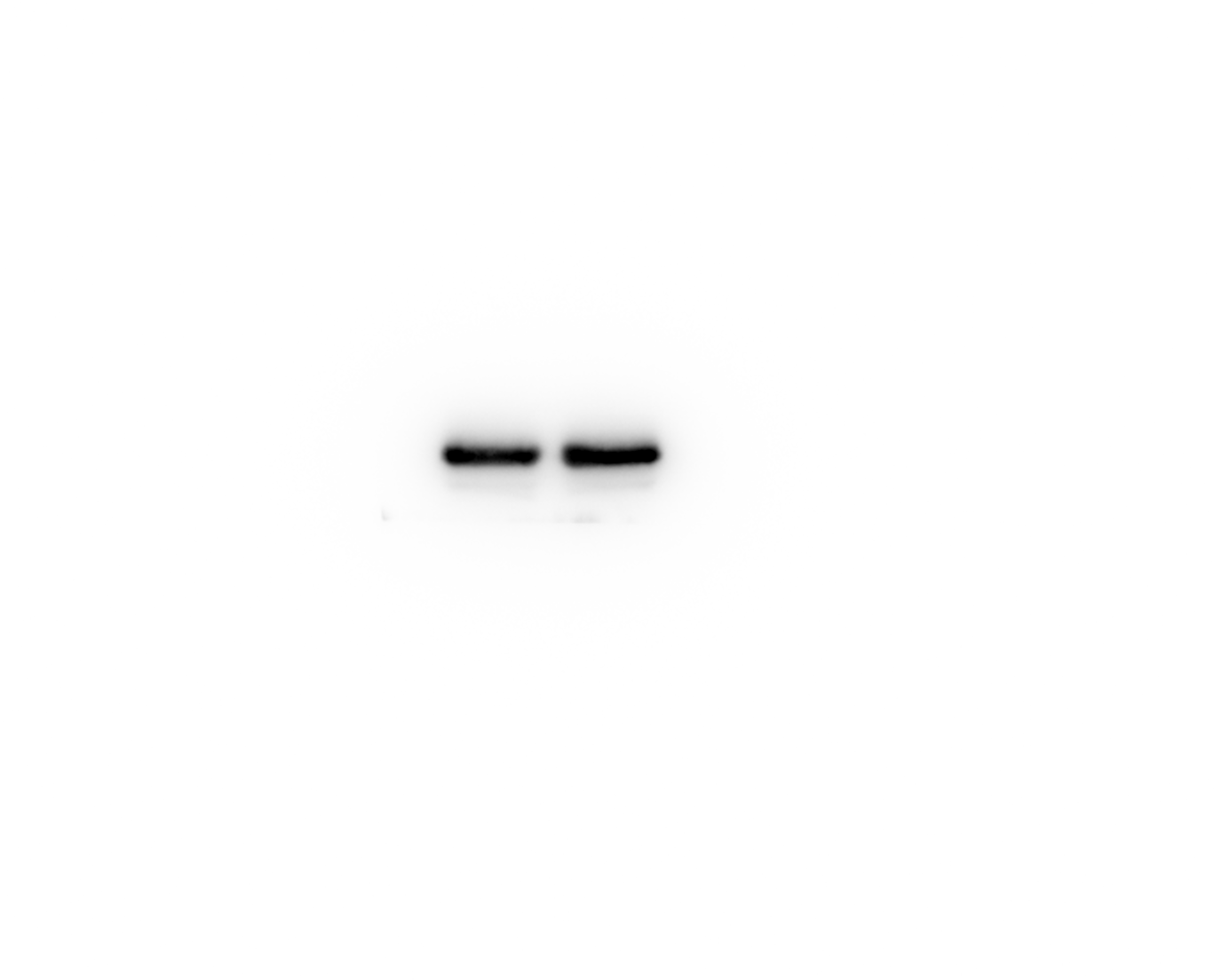

Supplement: Supplementary file 1 [file Supplementaryfile1.zip › Figure-Western blot images(revision-2025.05.22)/Figure 2. Western blot images/Figure2D.C4-2.β-tubulin.Tif]

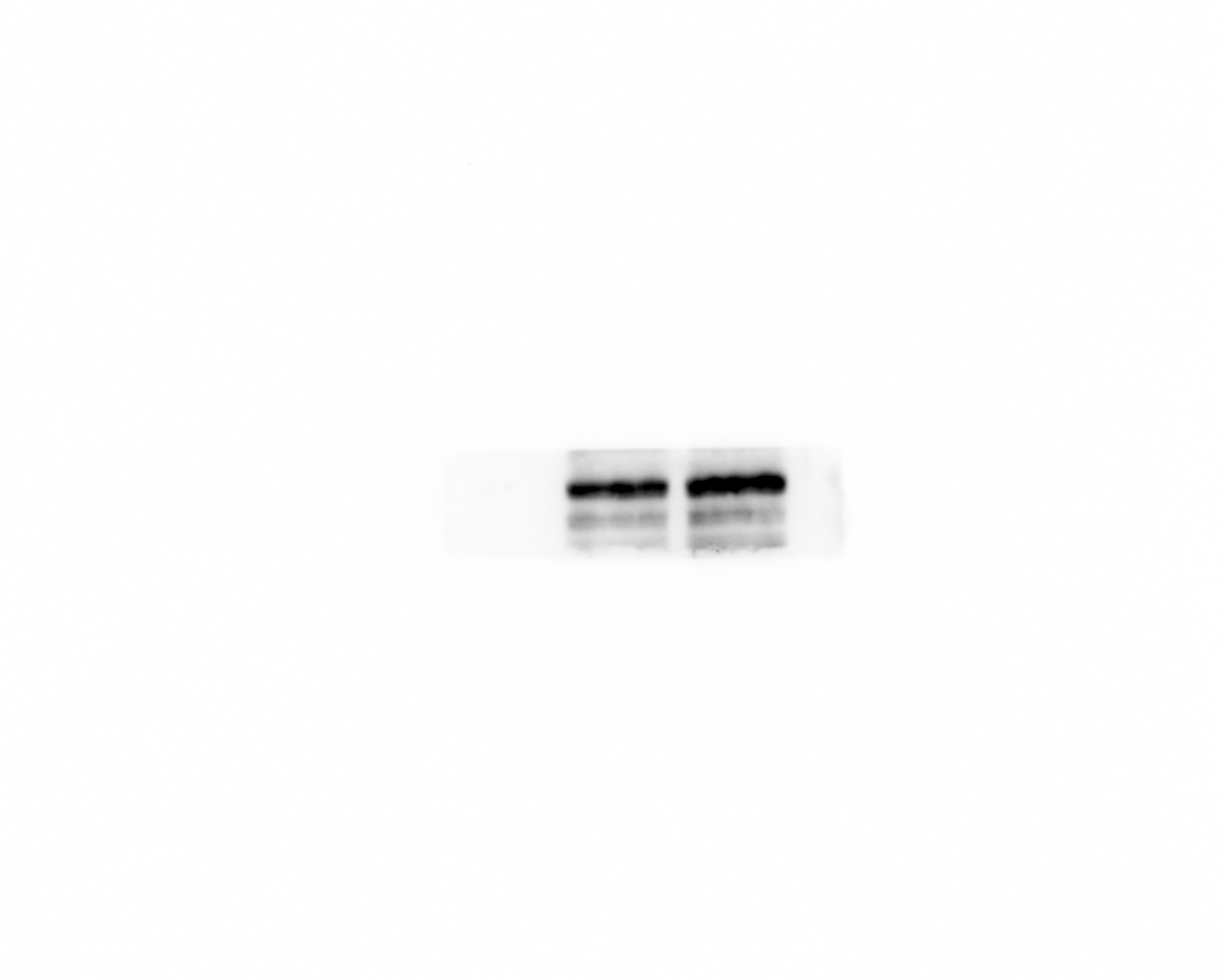

Supplement: Supplementary file 1 [file Supplementaryfile1.zip › Figure-Western blot images(revision-2025.05.22)/Figure 2. Western blot images/Figure2D.LNcap.AR.Tif]

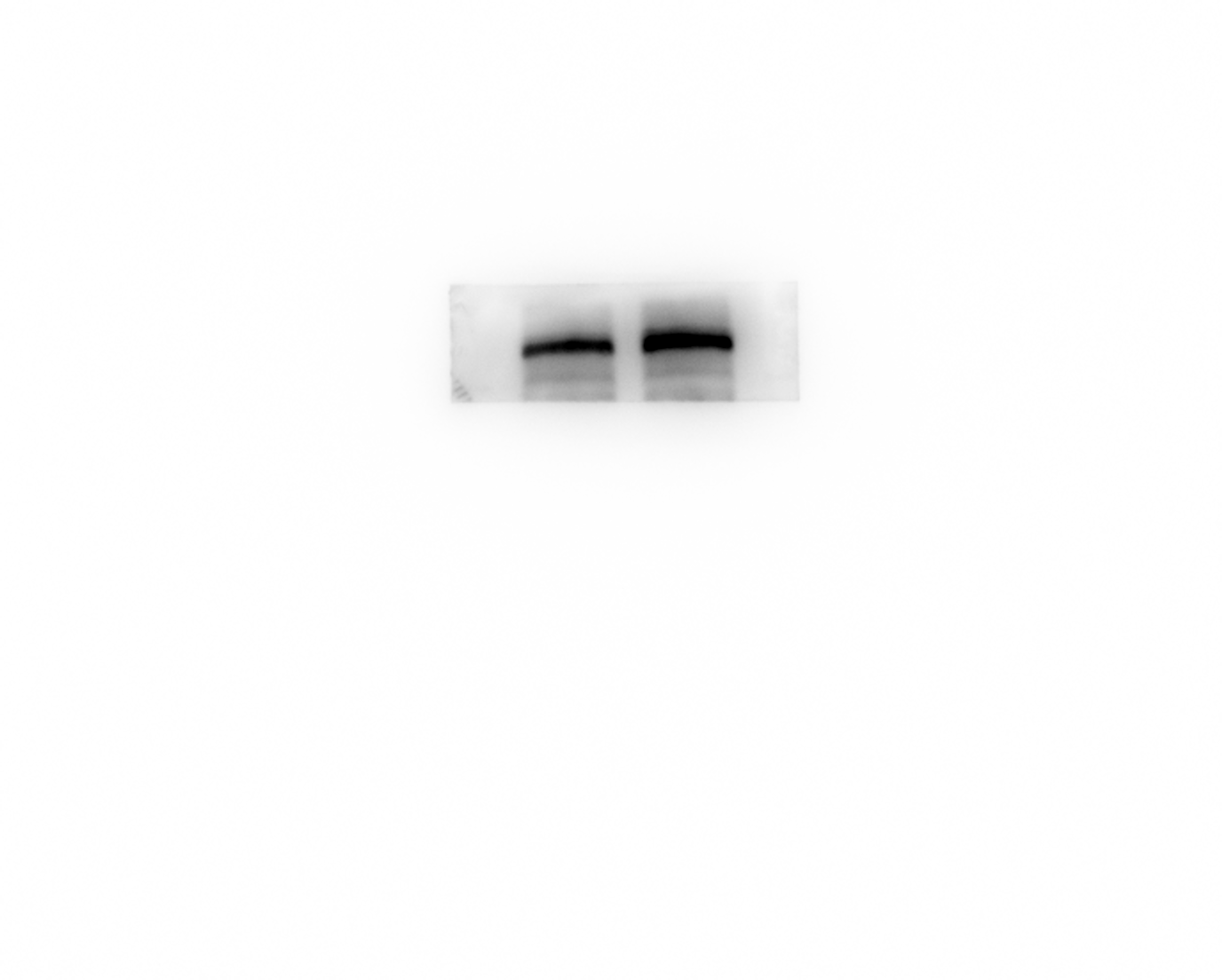

Supplement: Supplementary file 1 [file Supplementaryfile1.zip › Figure-Western blot images(revision-2025.05.22)/Figure 2. Western blot images/Figure2D.LNcap.ELOVL2.Tif]

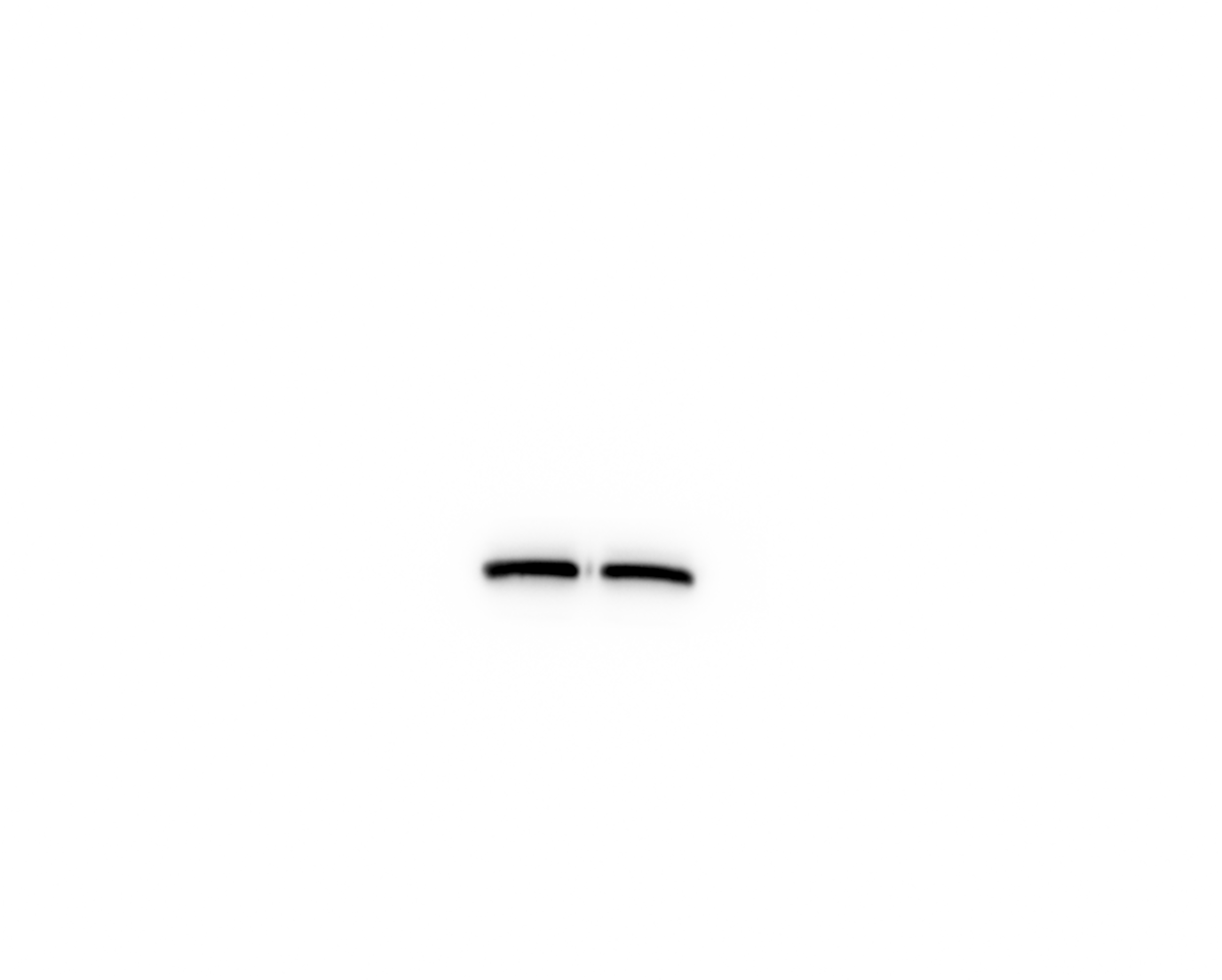

Supplement: Supplementary file 1 [file Supplementaryfile1.zip › Figure-Western blot images(revision-2025.05.22)/Figure 2. Western blot images/Figure2D.LNcap.β-tubulin.Tif]

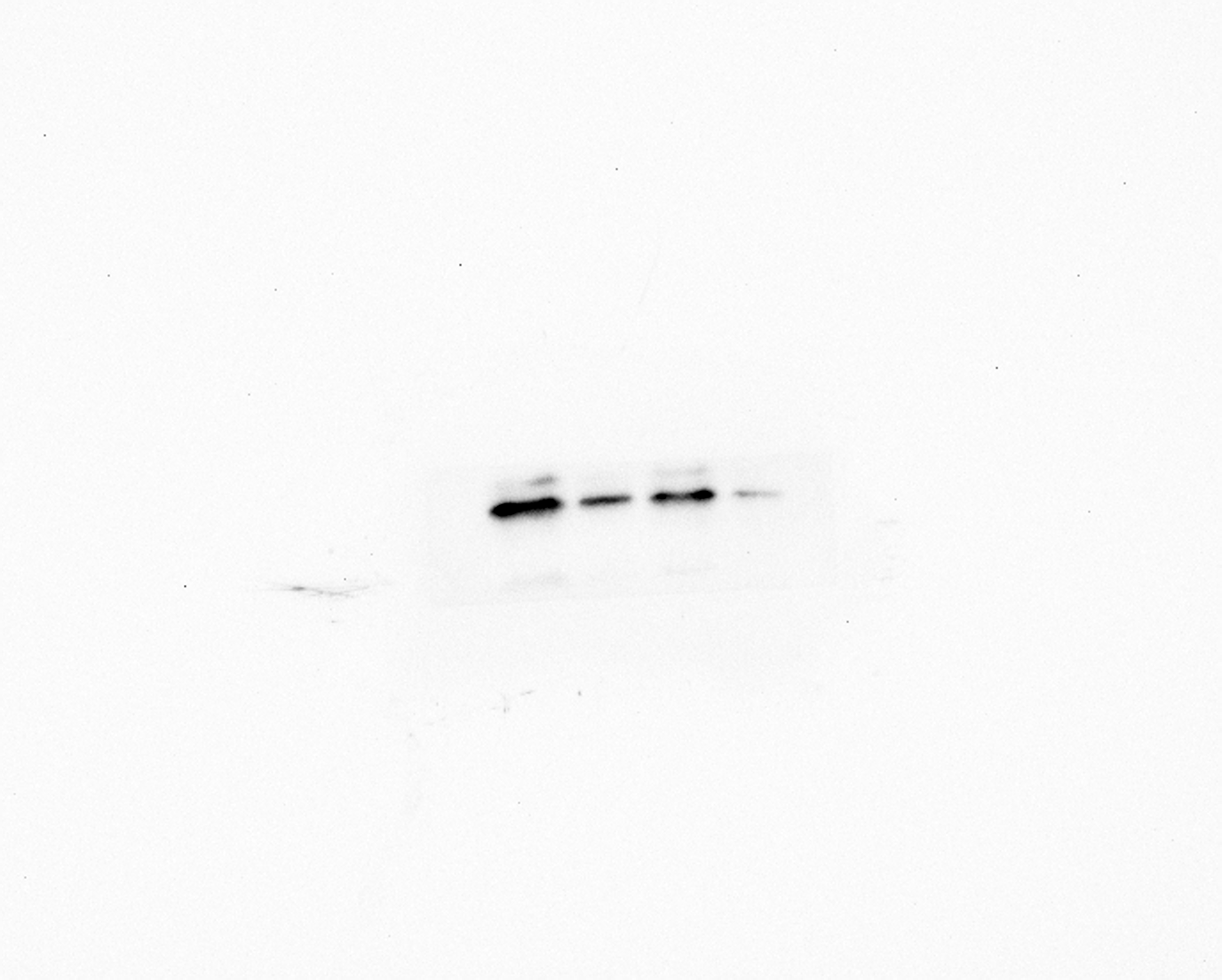

Supplement: Supplementary file 1 [file Supplementaryfile1.zip › Figure-Western blot images(revision-2025.05.22)/Figure 3. Western blot images/Figure3A.C4-2enZR.ELOVL2.Tif]

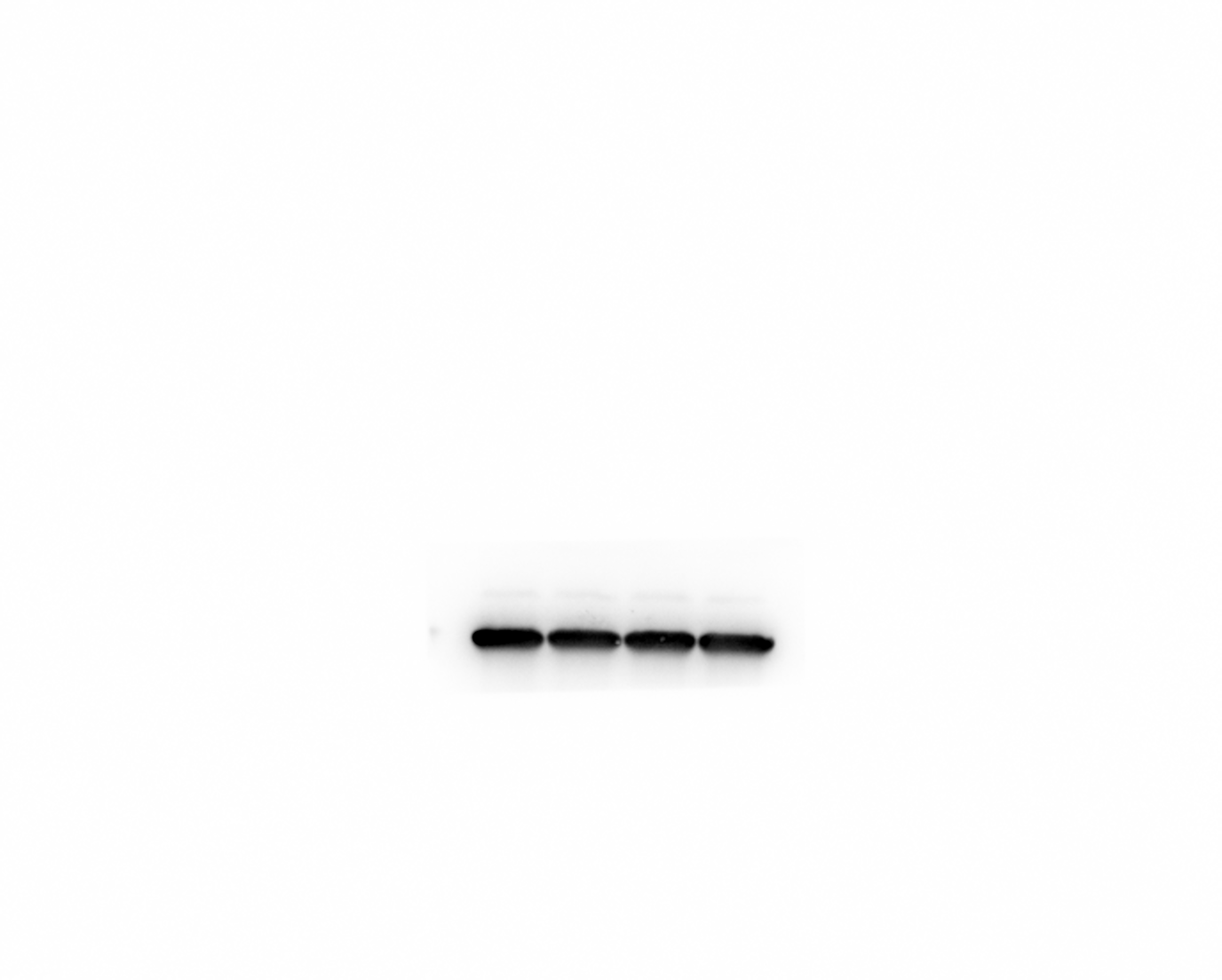

Supplement: Supplementary file 1 [file Supplementaryfile1.zip › Figure-Western blot images(revision-2025.05.22)/Figure 3. Western blot images/Figure3A.C4-2enZR.β-tubulin.Tif]

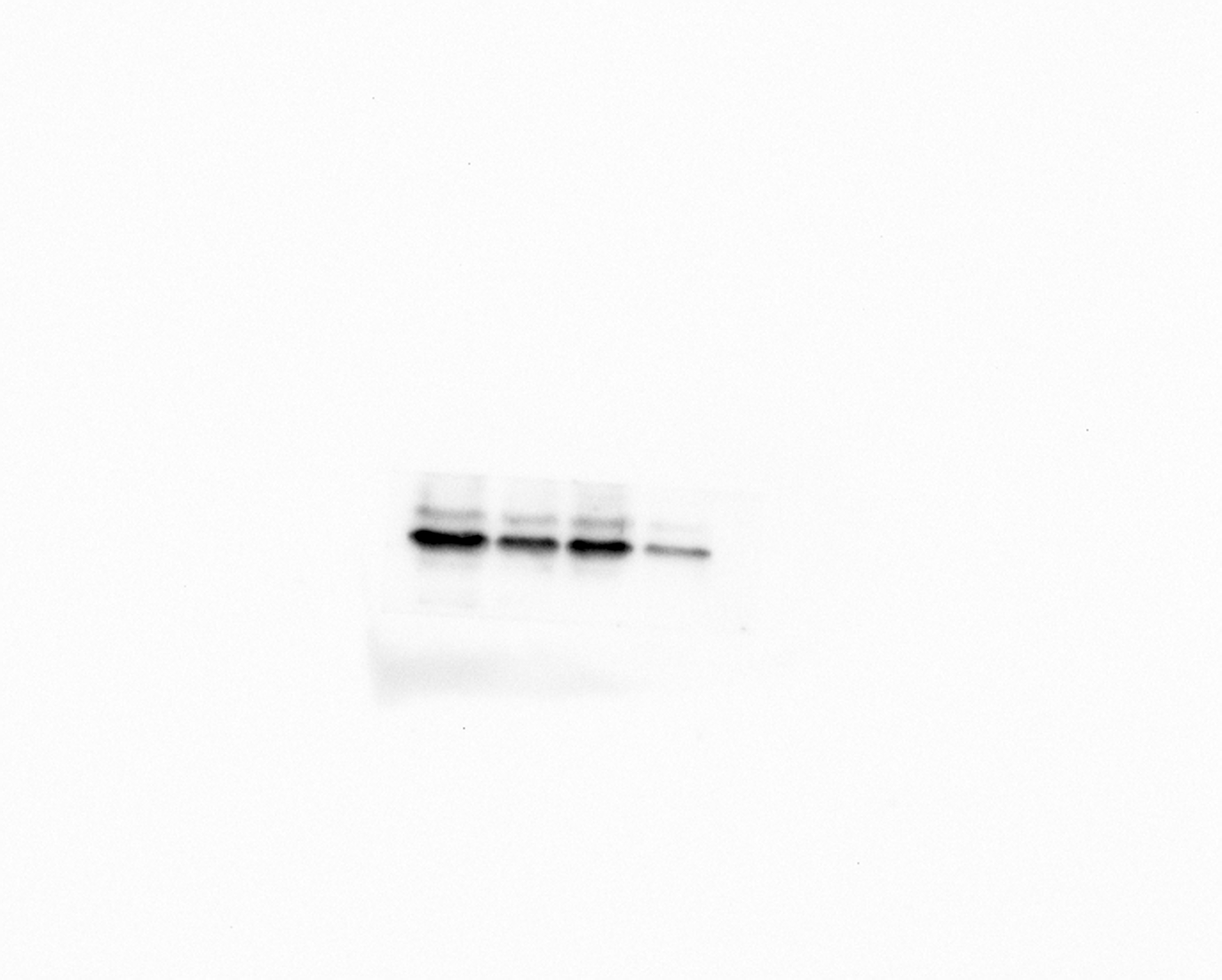

Supplement: Supplementary file 1 [file Supplementaryfile1.zip › Figure-Western blot images(revision-2025.05.22)/Figure 3. Western blot images/Figure3A.LNcap enZR.ELOVL2.Tif]

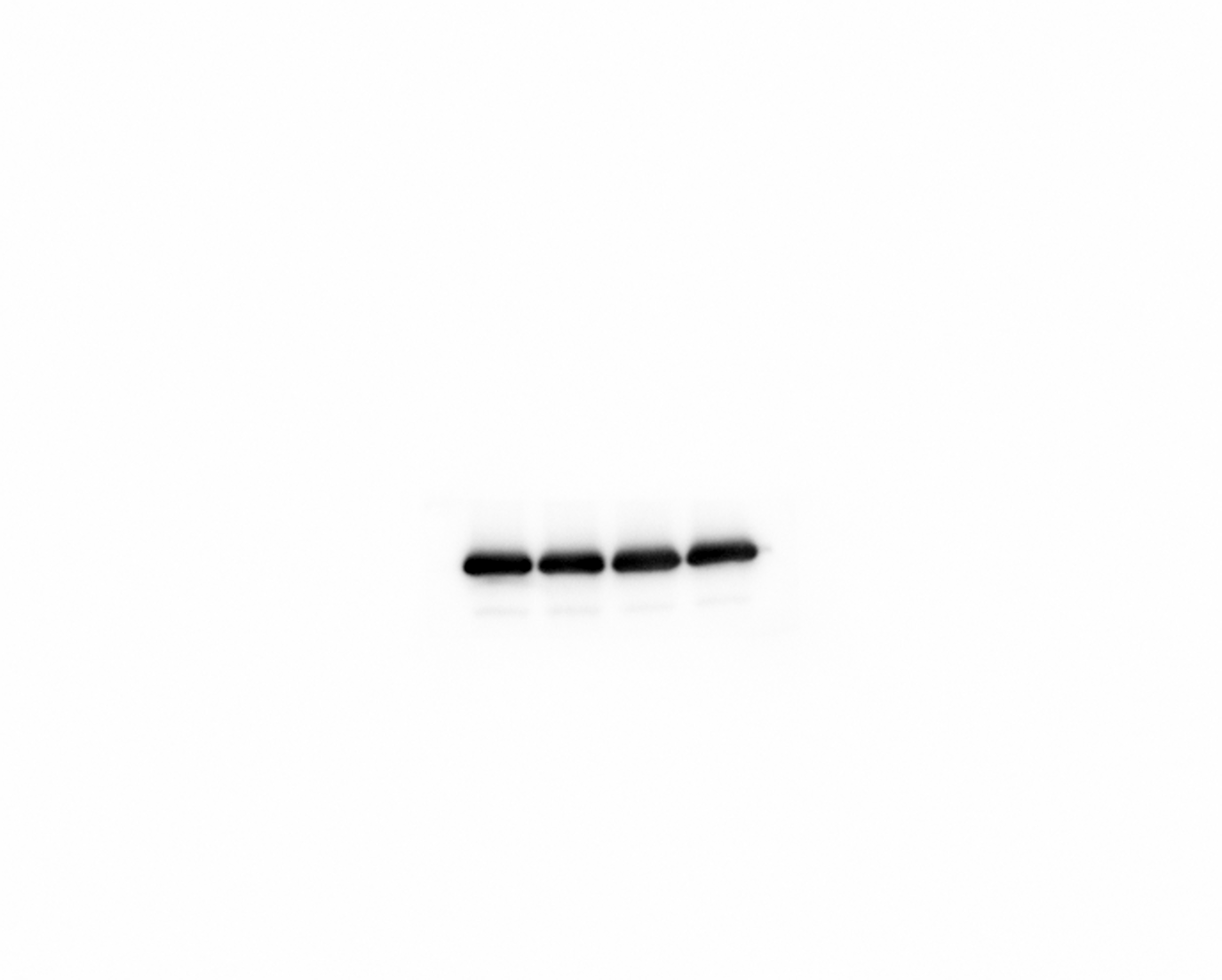

Supplement: Supplementary file 1 [file Supplementaryfile1.zip › Figure-Western blot images(revision-2025.05.22)/Figure 3. Western blot images/Figure3A.LNcap enZR.β-tubulin.Tif]

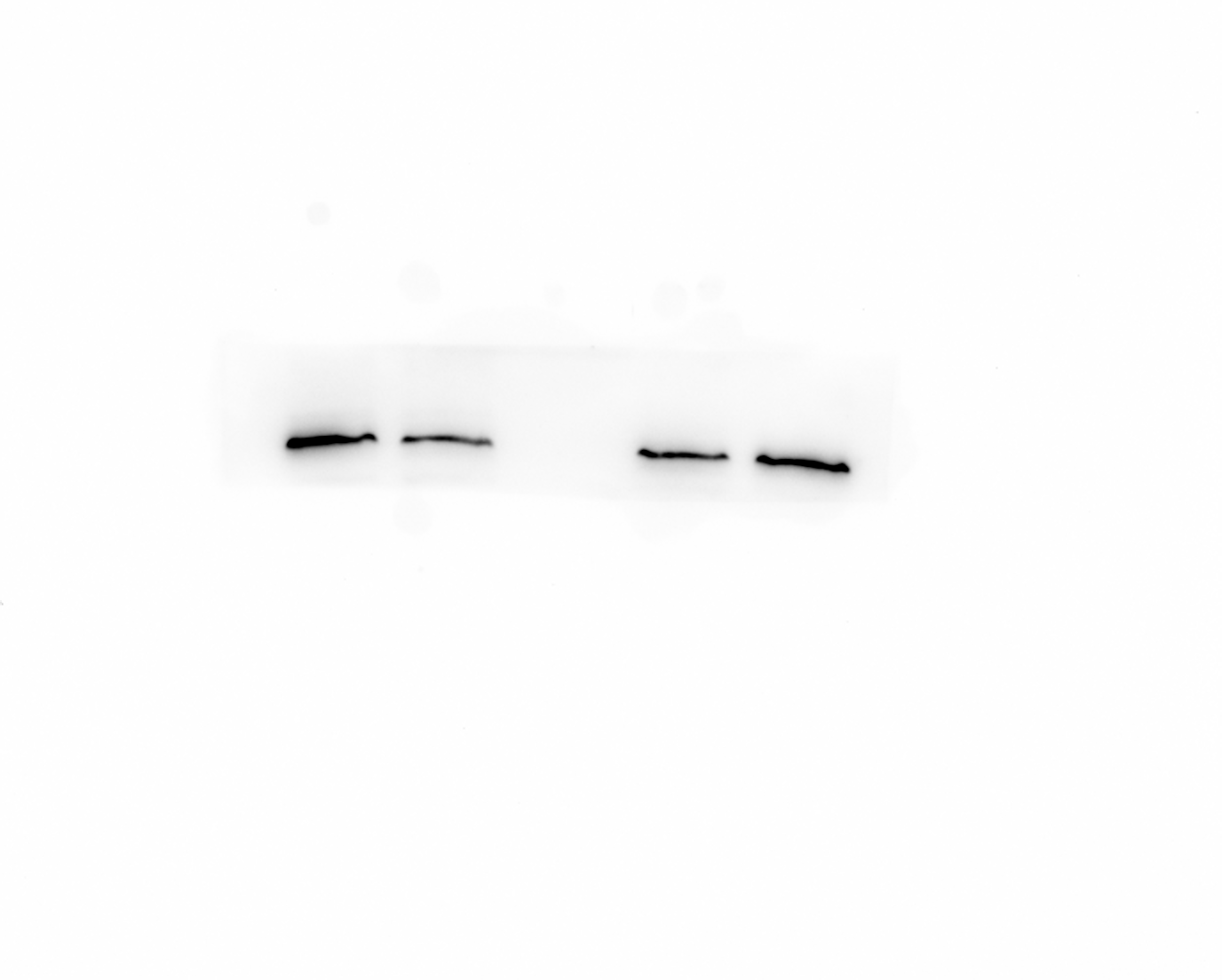

Supplement: Supplementary file 1 [file Supplementaryfile1.zip › Figure-Western blot images(revision-2025.05.22)/Figure 3. Western blot images/Figure3B.C4-2enZR.ELOVL2.Tif]

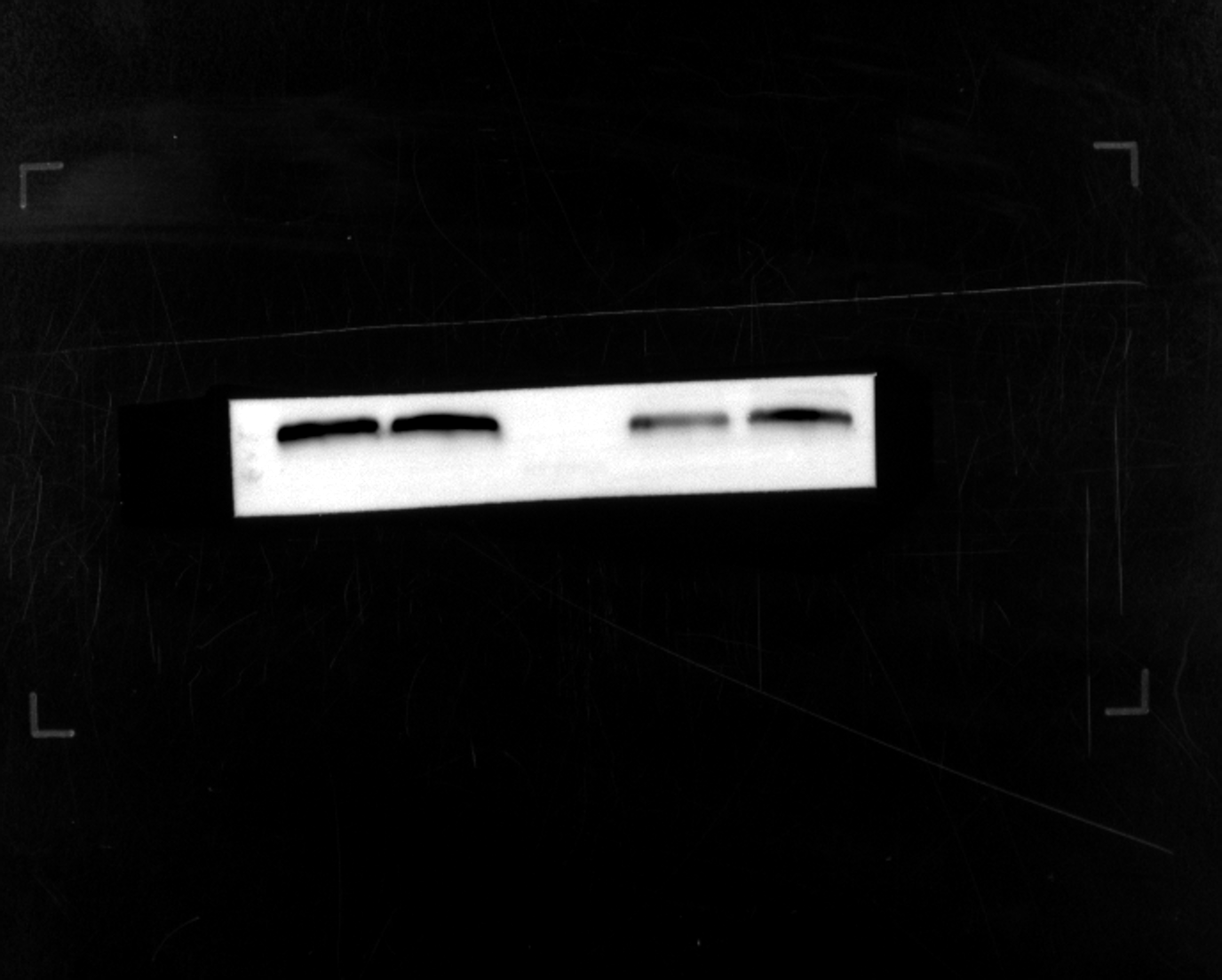

Supplement: Supplementary file 1 [file Supplementaryfile1.zip › Figure-Western blot images(revision-2025.05.22)/Figure 3. Western blot images/Figure3B.C4-2enZR.β-tubulin.Tif]

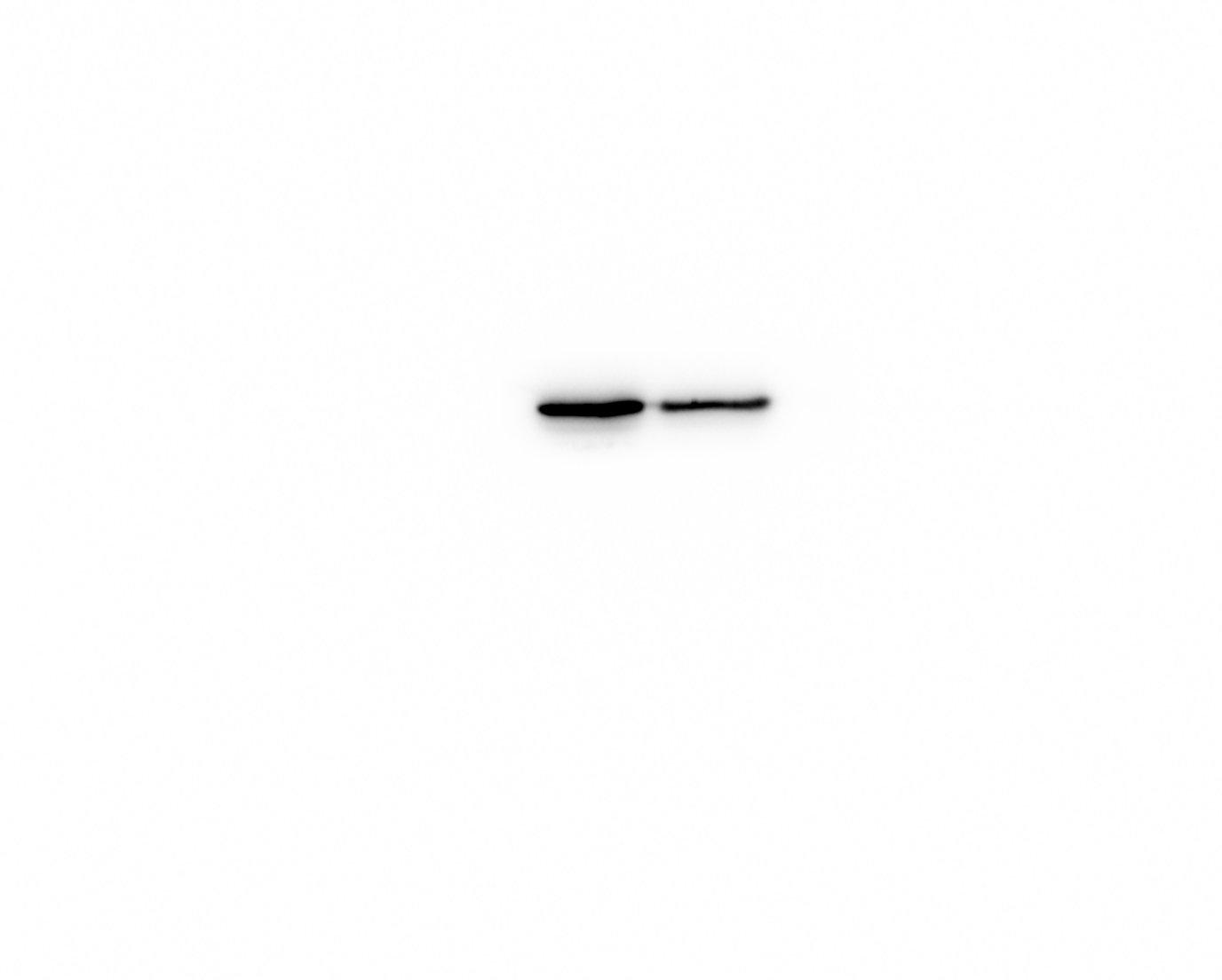

Supplement: Supplementary file 1 [file Supplementaryfile1.zip › Figure-Western blot images(revision-2025.05.22)/Figure 3. Western blot images/Figure3B.LNcap enZR.ELOVL2.Tif]

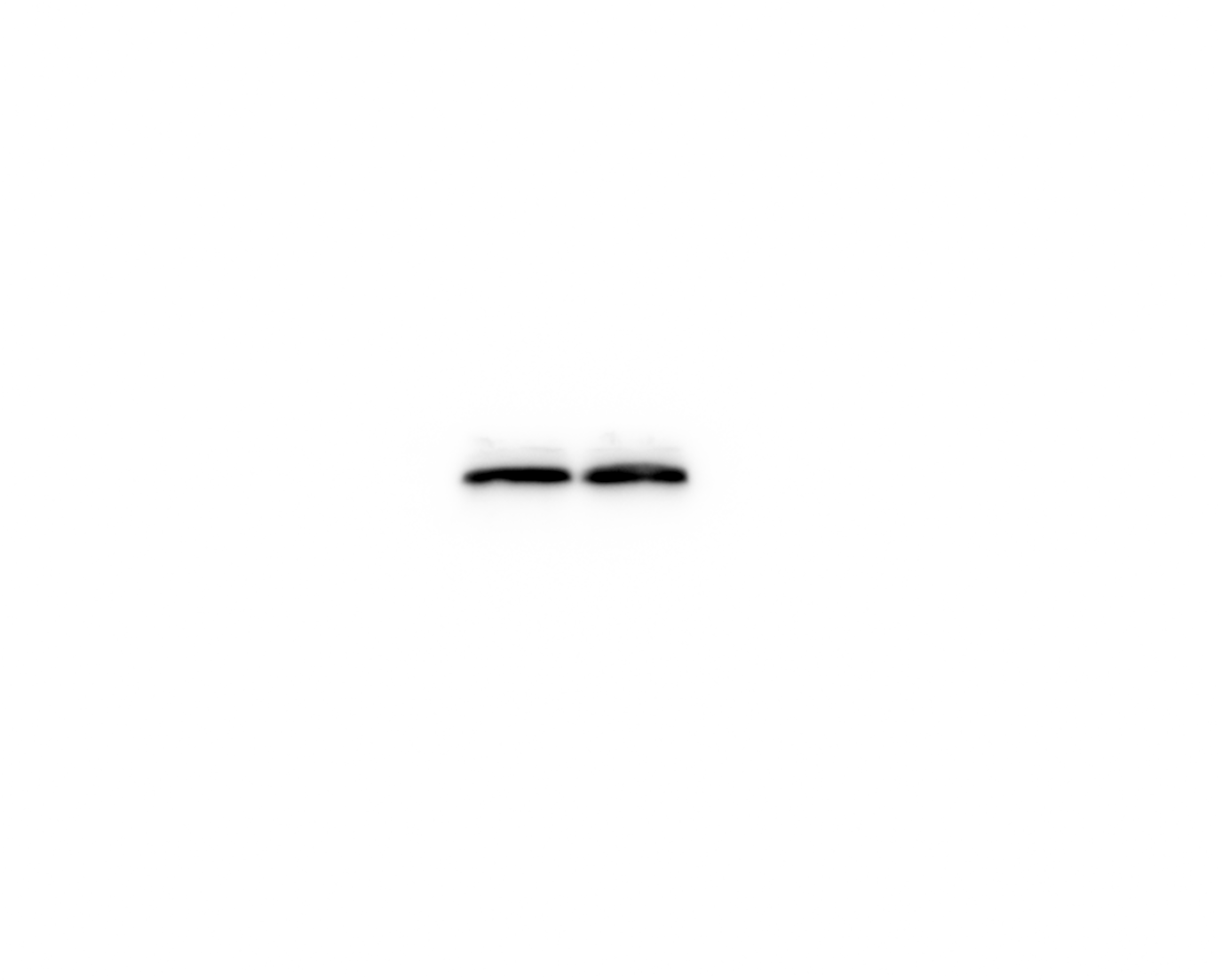

Supplement: Supplementary file 1 [file Supplementaryfile1.zip › Figure-Western blot images(revision-2025.05.22)/Figure 3. Western blot images/Figure3B.LNcap enZR.β-tubulin.Tif]

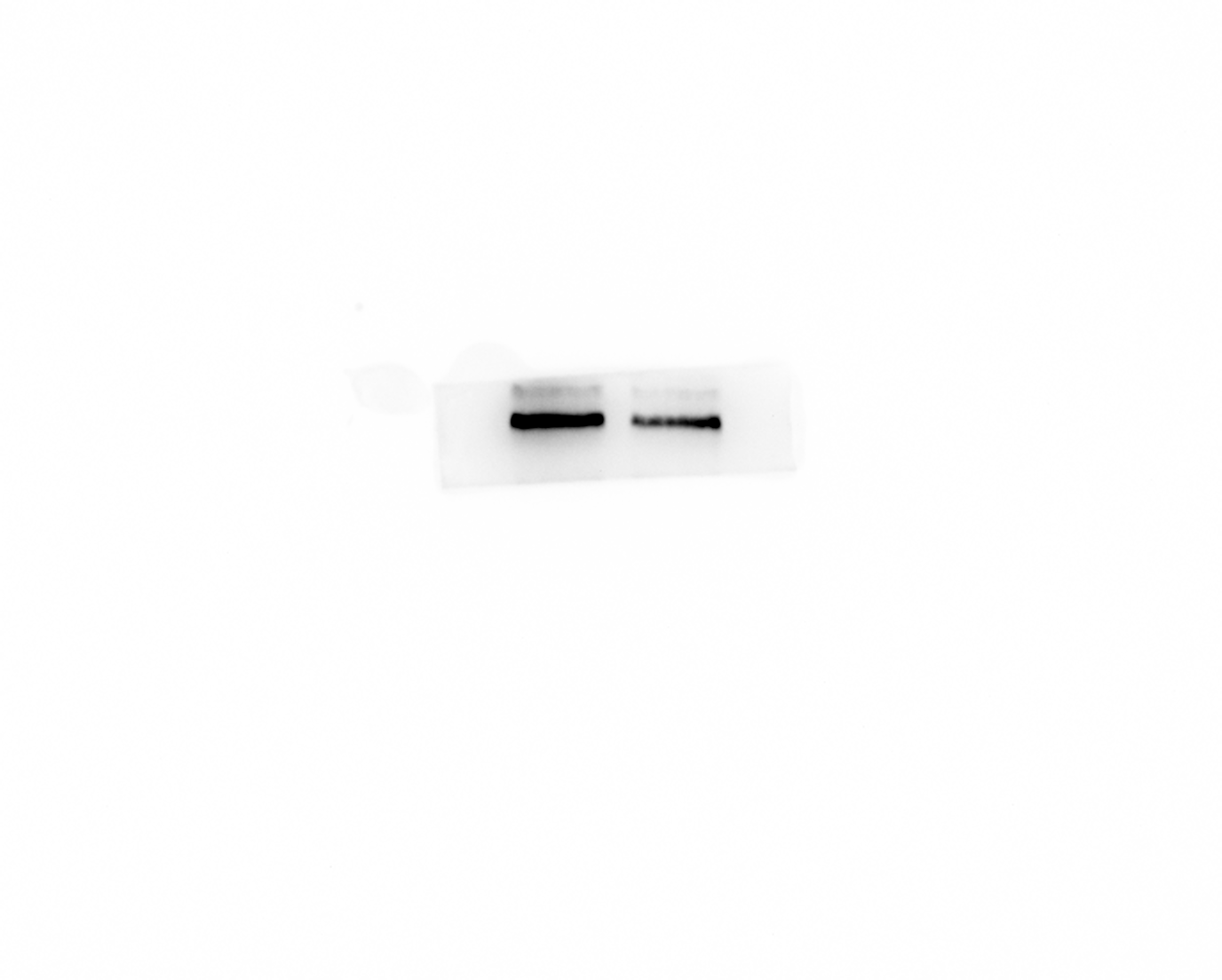

Supplement: Supplementary file 1 [file Supplementaryfile1.zip › Figure-Western blot images(revision-2025.05.22)/Figure 4. Western blot images/Figure4B.C4-2 enZR.AR.Tif]

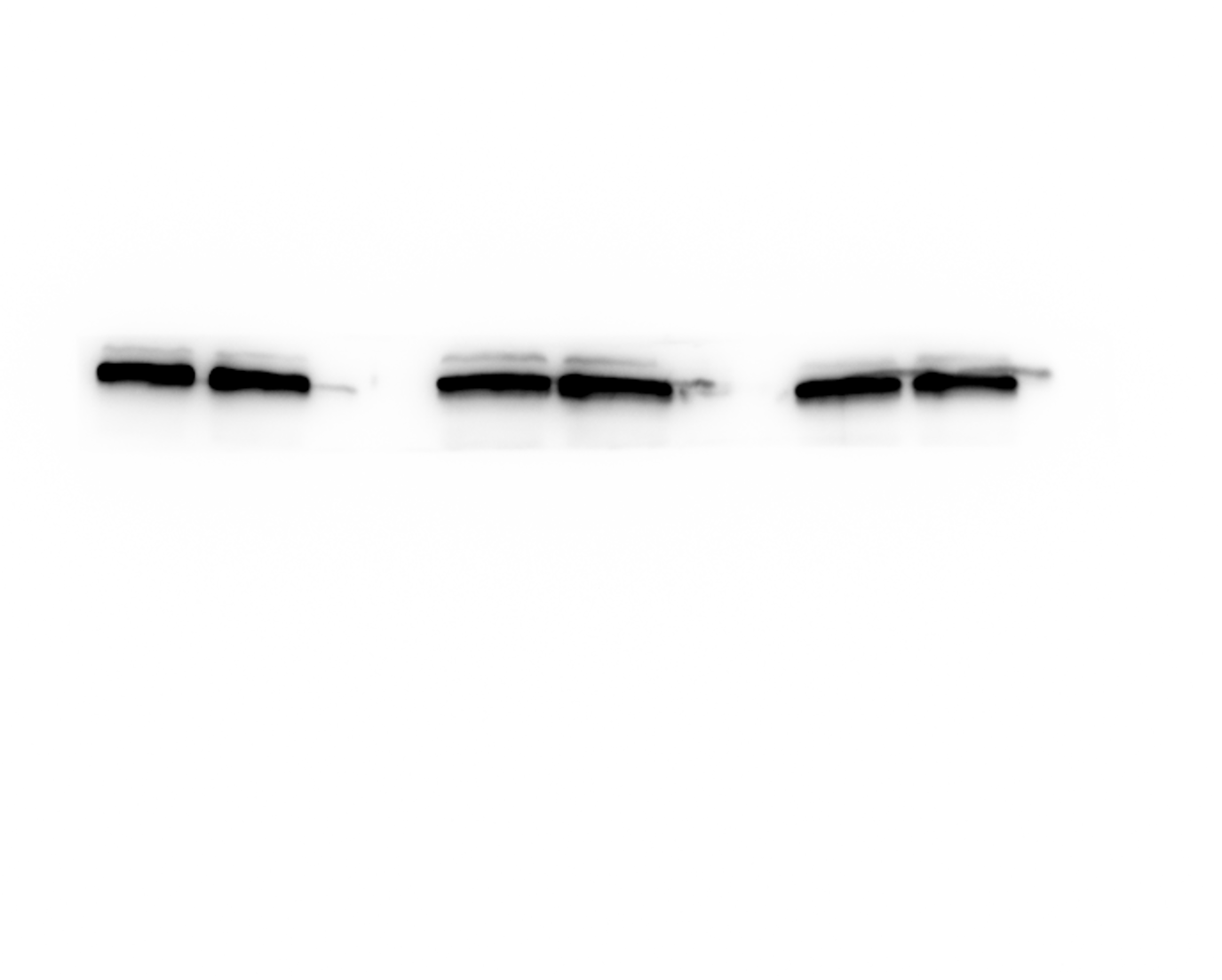

Supplement: Supplementary file 1 [file Supplementaryfile1.zip › Figure-Western blot images(revision-2025.05.22)/Figure 4. Western blot images/Figure4B.C4-2 enZR.β-tubulin(left-1).Tif]

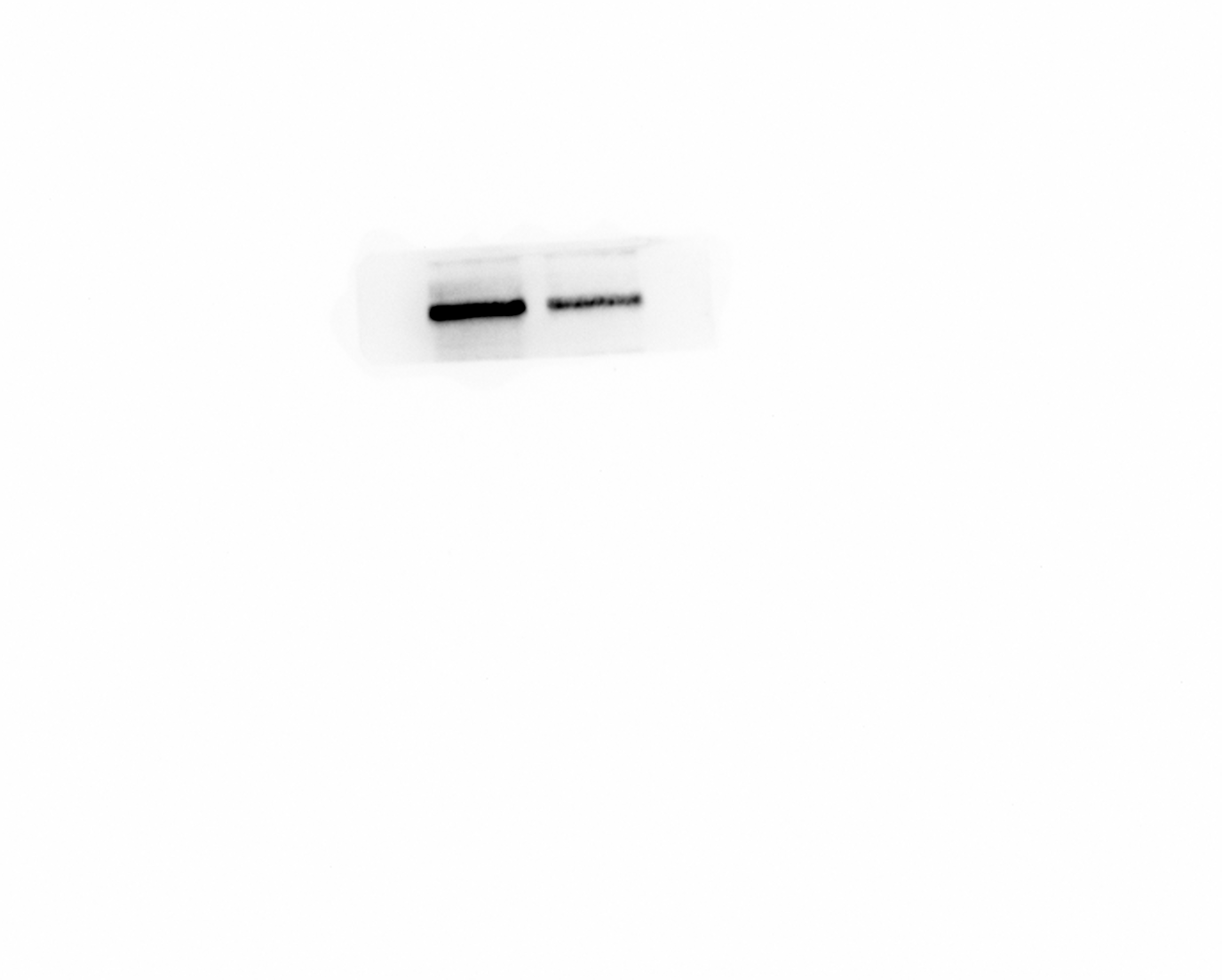

Supplement: Supplementary file 1 [file Supplementaryfile1.zip › Figure-Western blot images(revision-2025.05.22)/Figure 4. Western blot images/Figure4B.LNcap enZR.AR.Tif]

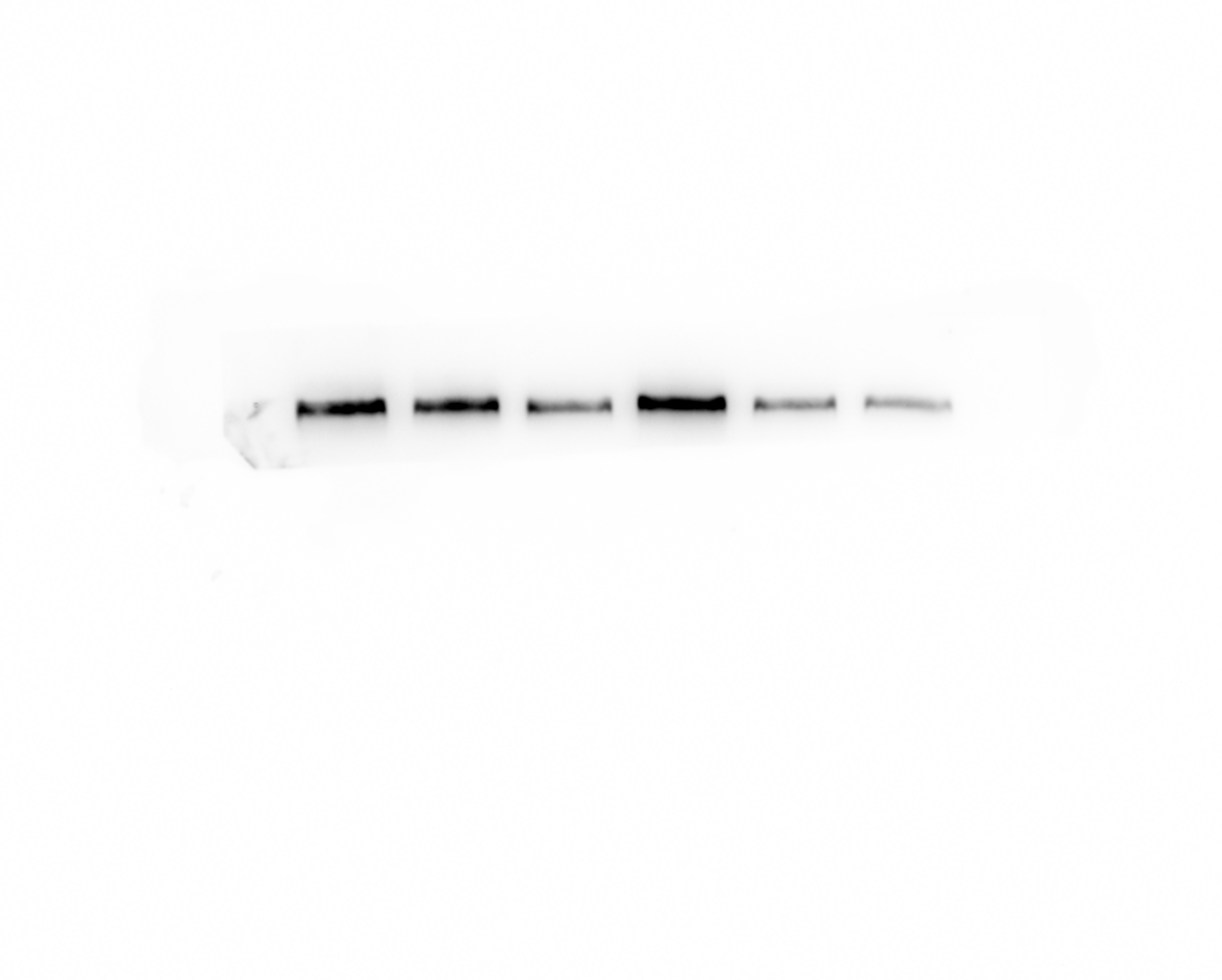

Supplement: Supplementary file 1 [file Supplementaryfile1.zip › Figure-Western blot images(revision-2025.05.22)/Figure 4. Western blot images/Figure4C.C4-2 enZR.AR(left- NC, right-sh-ELOVL2).Tif]

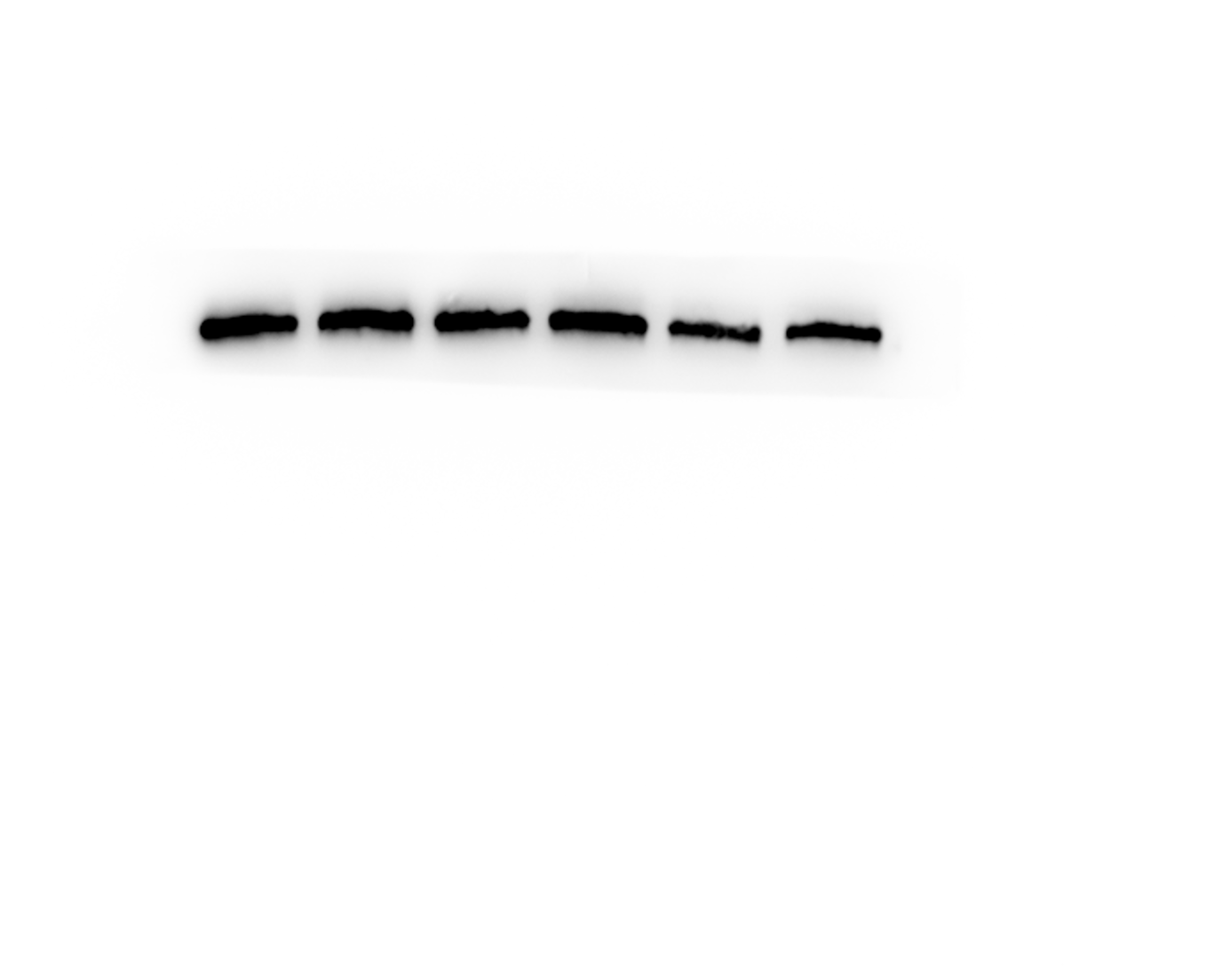

Supplement: Supplementary file 1 [file Supplementaryfile1.zip › Figure-Western blot images(revision-2025.05.22)/Figure 4. Western blot images/Figure4C.C4-2 enZR.β-tubulin(left- NC, right-sh-ELOVL2).Tif]

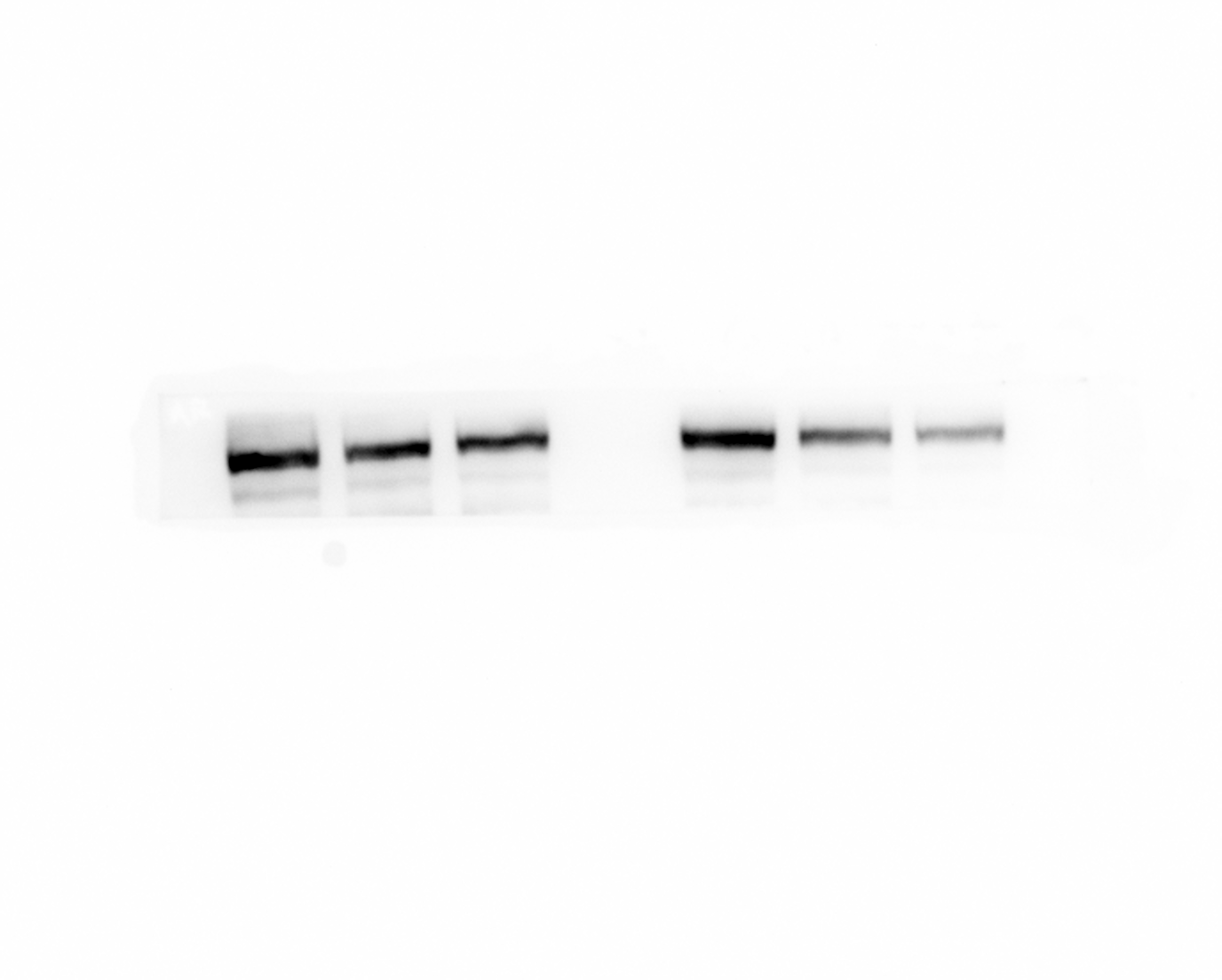

Supplement: Supplementary file 1 [file Supplementaryfile1.zip › Figure-Western blot images(revision-2025.05.22)/Figure 4. Western blot images/Figure4C.LNcap enZR.AR(left- NC, right-sh-ELOVL2).Tif]

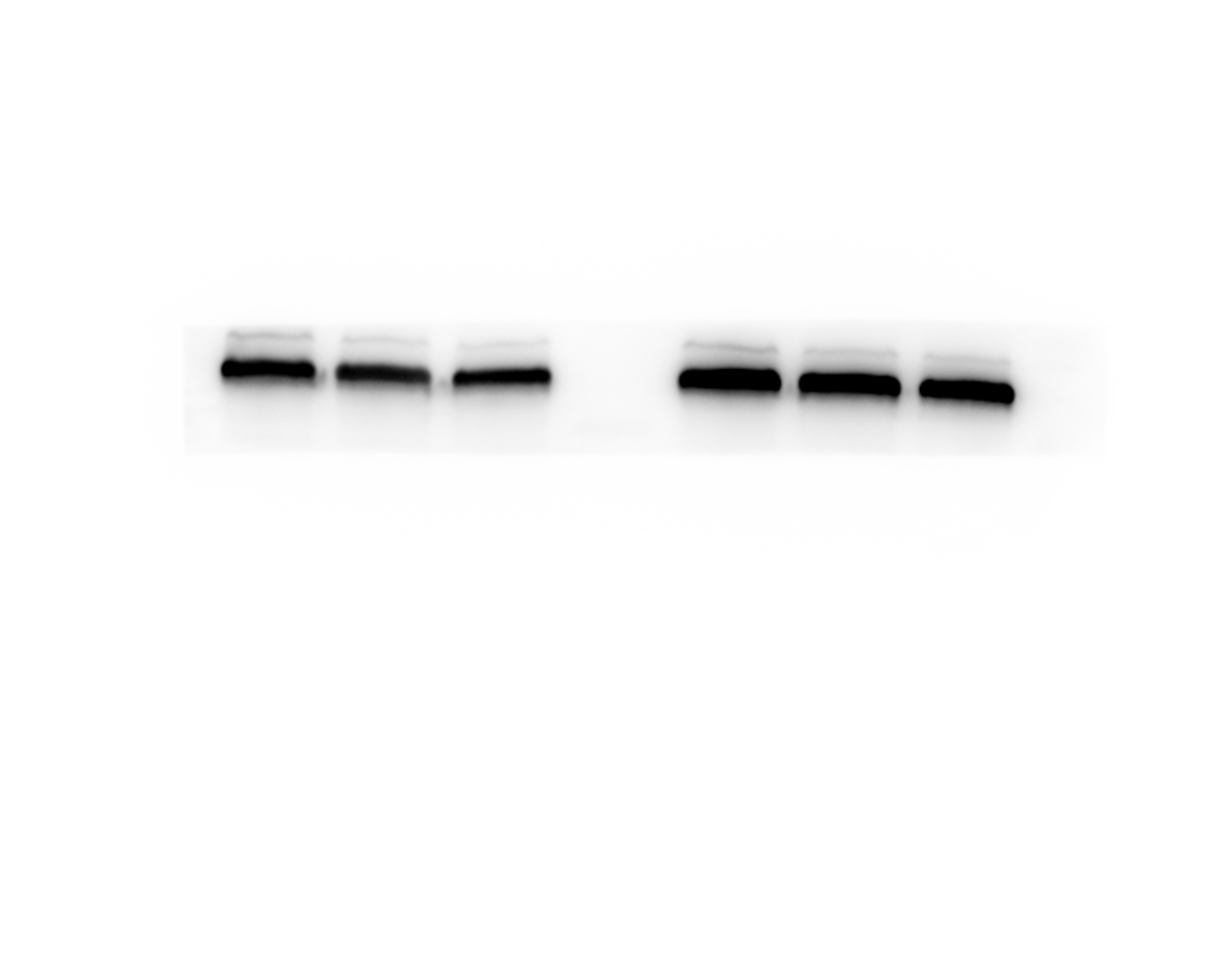

Supplement: Supplementary file 1 [file Supplementaryfile1.zip › Figure-Western blot images(revision-2025.05.22)/Figure 4. Western blot images/Figure4C.LNcap enZR.β-tubulin((left- NC, right-sh-ELOVL2)).Tif]

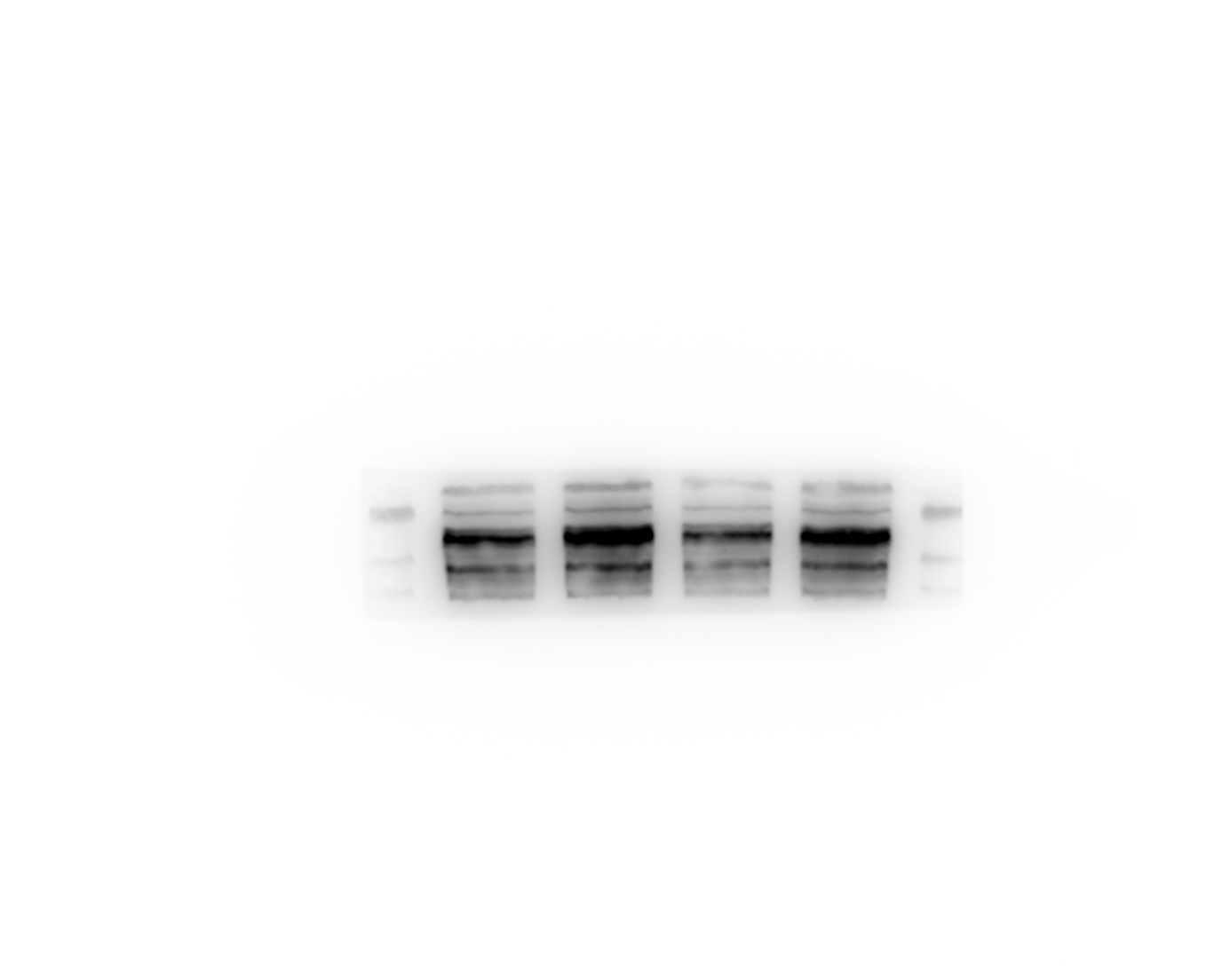

Supplement: Supplementary file 1 [file Supplementaryfile1.zip › Figure-Western blot images(revision-2025.05.22)/Figure 4. Western blot images/Figure4D.C4-2 enZR.AR.Tif]

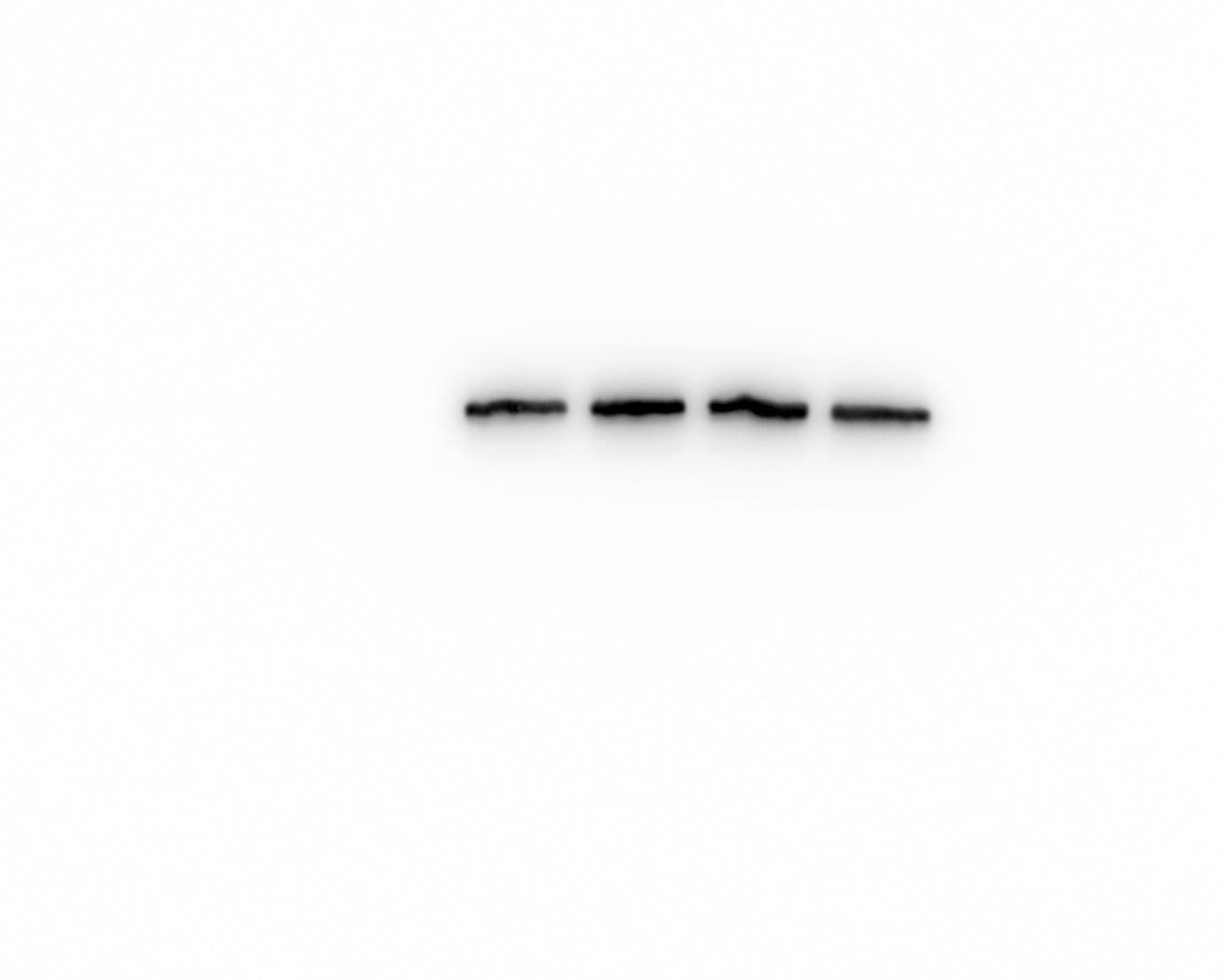

Supplement: Supplementary file 1 [file Supplementaryfile1.zip › Figure-Western blot images(revision-2025.05.22)/Figure 4. Western blot images/Figure4D.C4-2 enZR.β-tubulin.Tif]

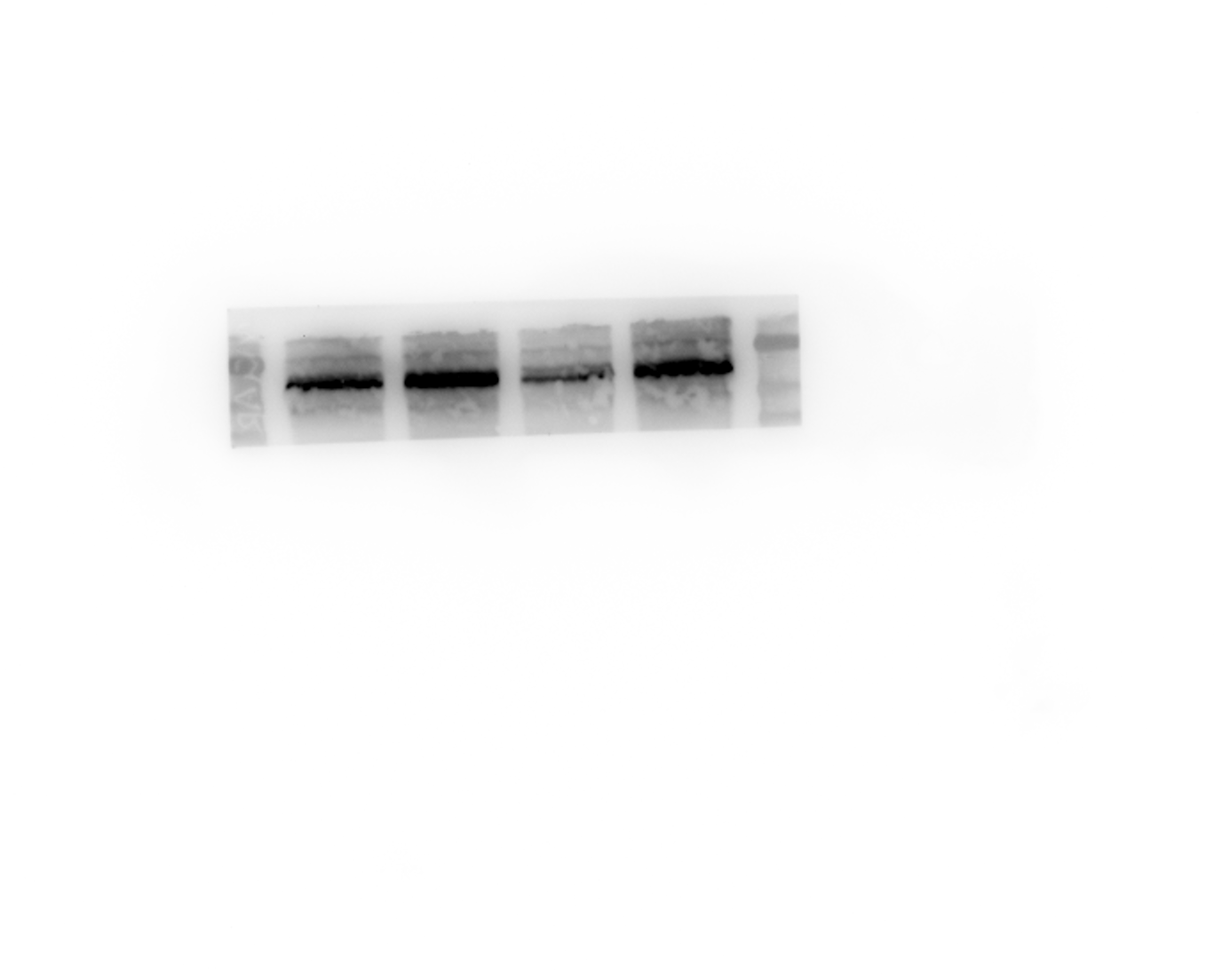

Supplement: Supplementary file 1 [file Supplementaryfile1.zip › Figure-Western blot images(revision-2025.05.22)/Figure 4. Western blot images/Figure4D.LNcap enZR.AR.Tif]

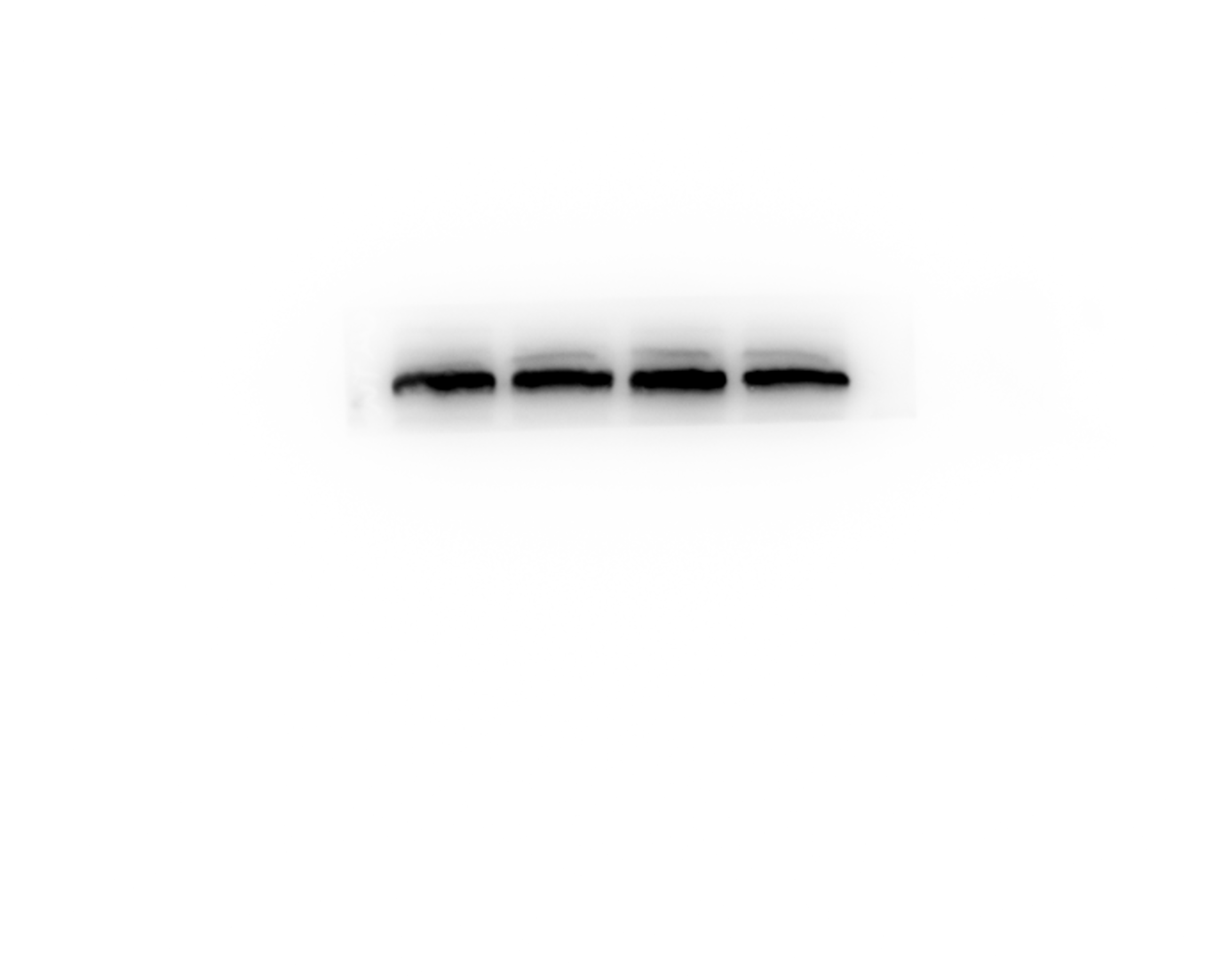

Supplement: Supplementary file 1 [file Supplementaryfile1.zip › Figure-Western blot images(revision-2025.05.22)/Figure 4. Western blot images/Figure4D.LNcap enZR.β-tubulin.Tif]

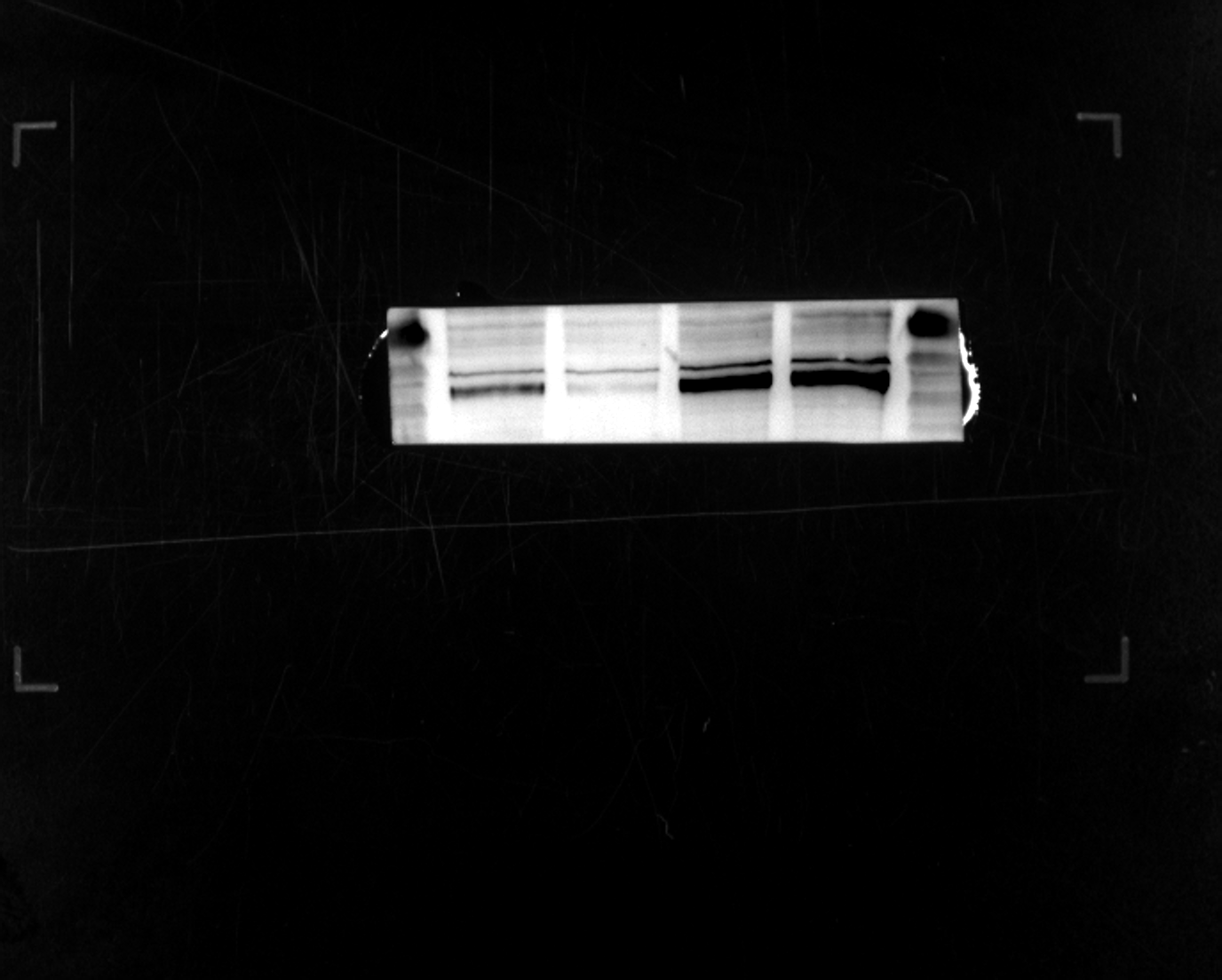

Supplement: Supplementary file 1 [file Supplementaryfile1.zip › Figure-Western blot images(revision-2025.05.22)/Figure 4. Western blot images/Figure4E.C4-2 enZR.AR.Tif]

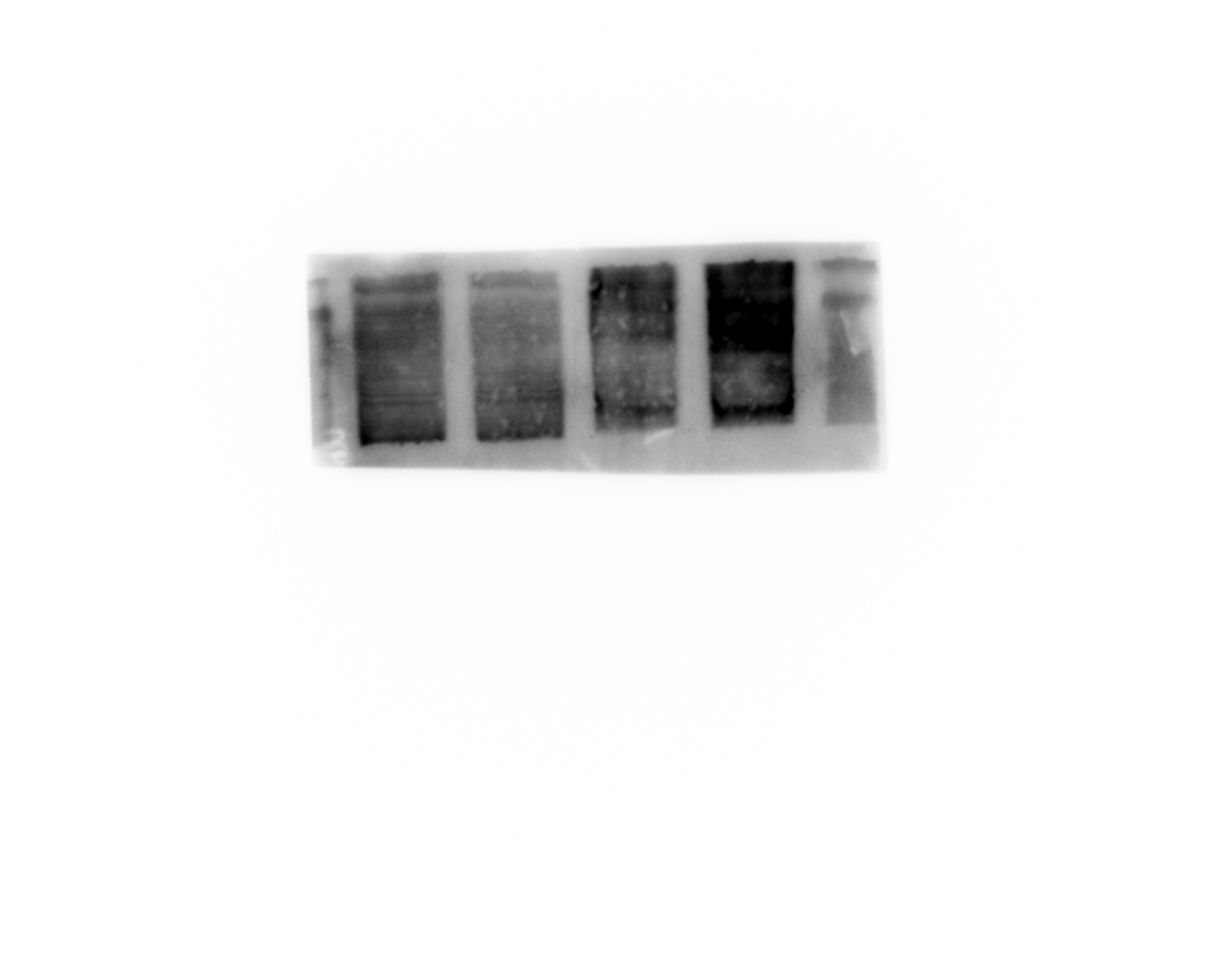

Supplement: Supplementary file 1 [file Supplementaryfile1.zip › Figure-Western blot images(revision-2025.05.22)/Figure 4. Western blot images/Figure4E.C4-2 enZR.Ubiquitin.Tif]

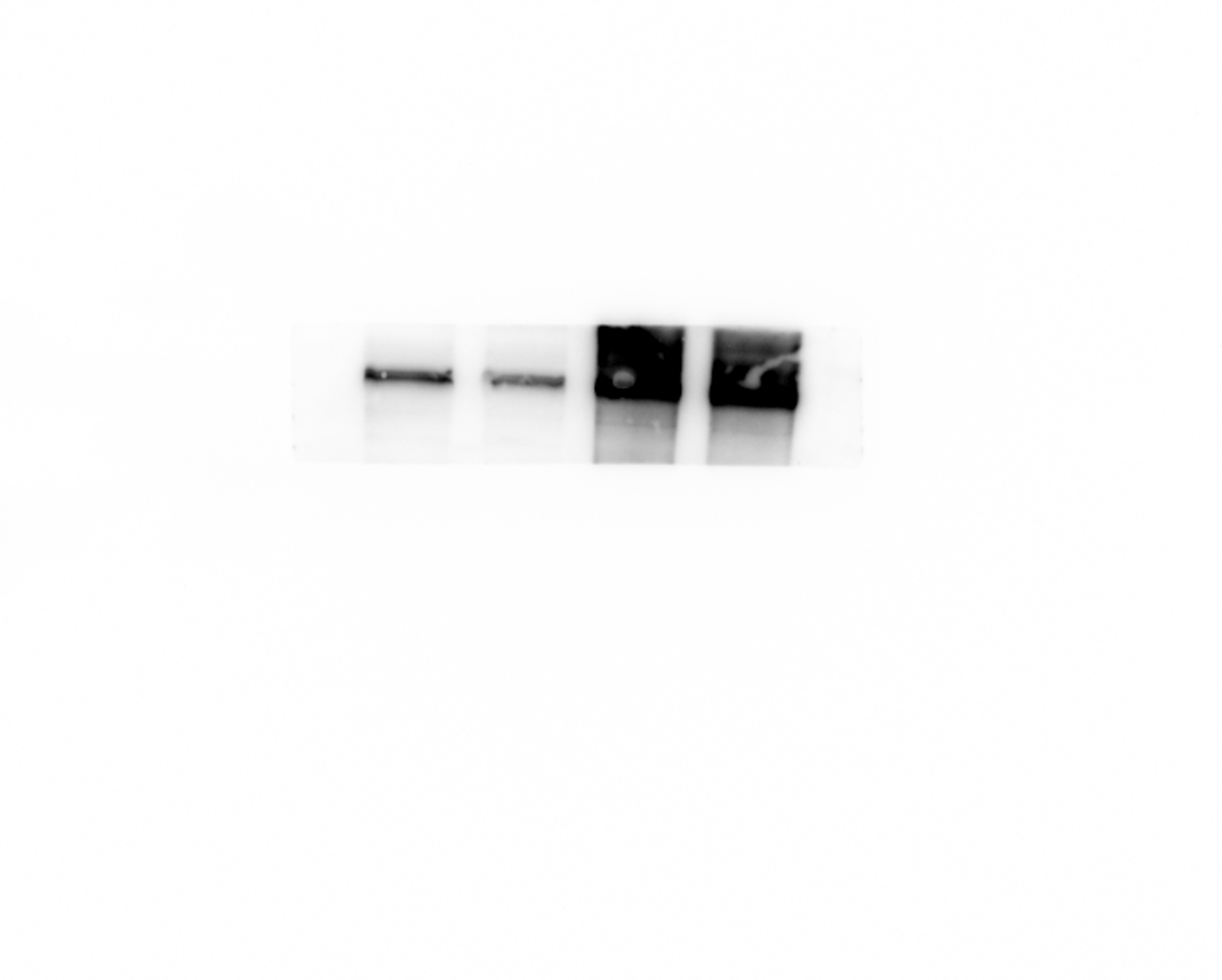

Supplement: Supplementary file 1 [file Supplementaryfile1.zip › Figure-Western blot images(revision-2025.05.22)/Figure 4. Western blot images/Figure4E.LNcap enZR.AR.Tif]

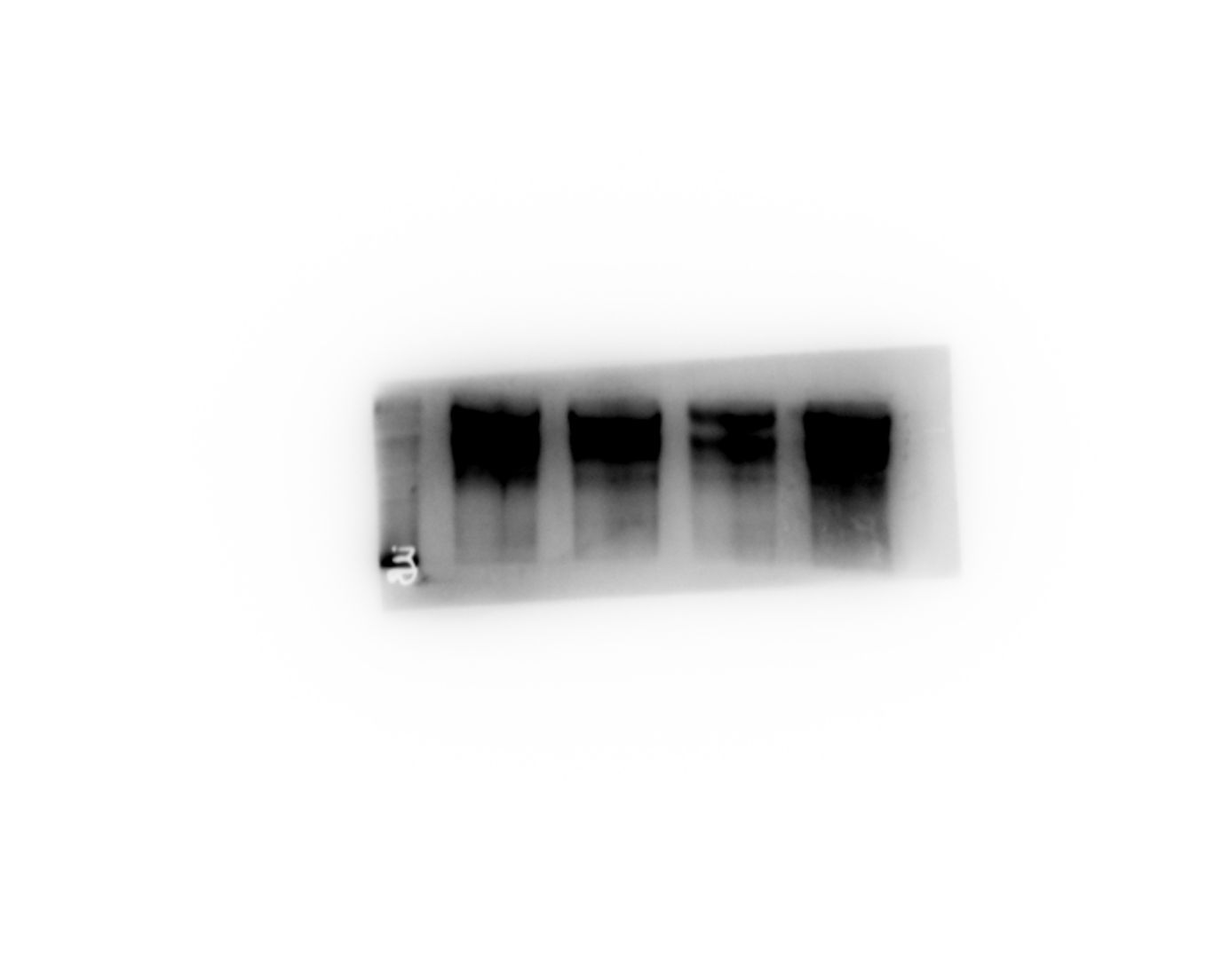

Supplement: Supplementary file 1 [file Supplementaryfile1.zip › Figure-Western blot images(revision-2025.05.22)/Figure 4. Western blot images/Figure4E.LNcap enZR.Ubiquitin.Tif]

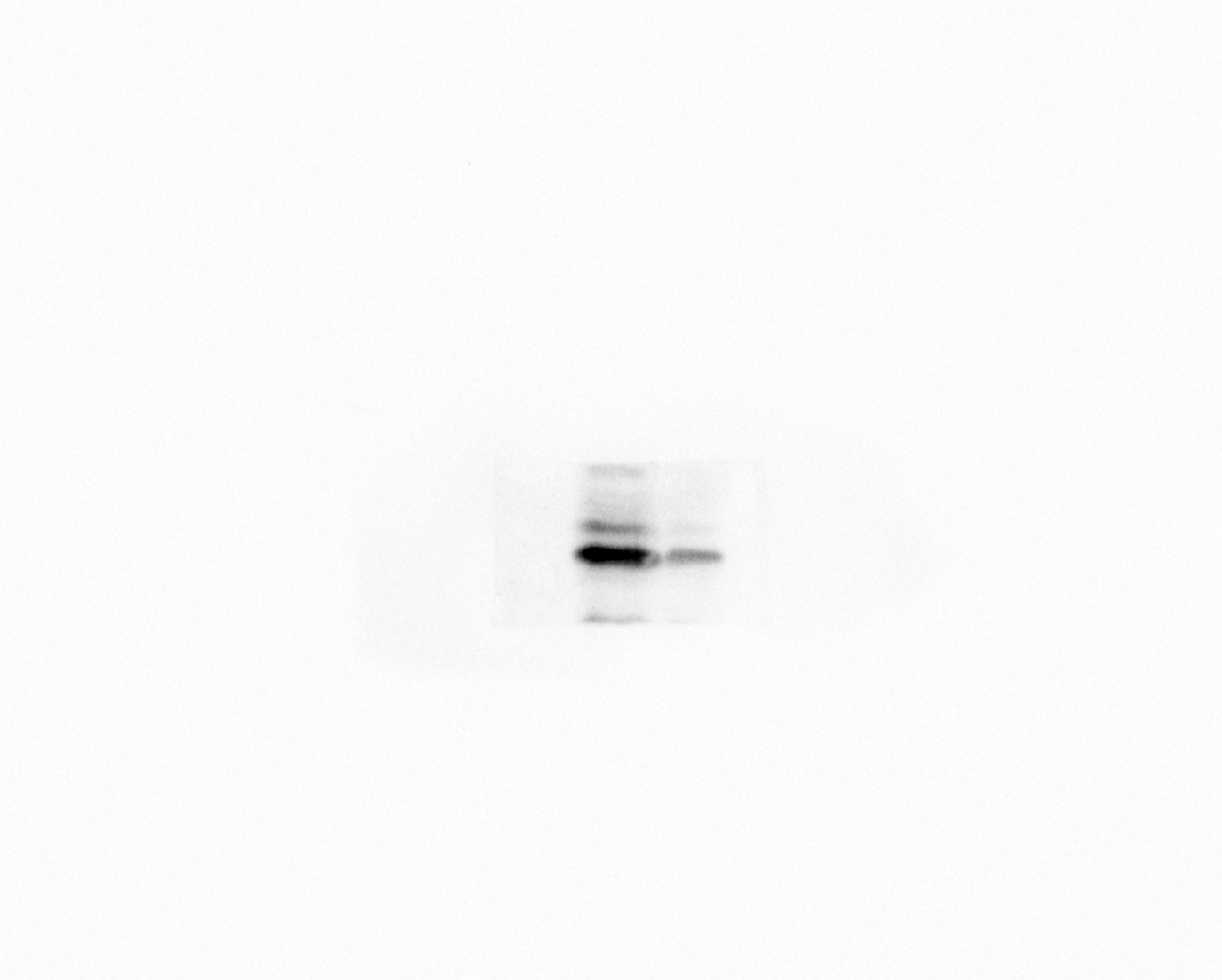

Supplement: Supplementary file 1 [file Supplementaryfile1.zip › Figure-Western blot images(revision-2025.05.22)/Figure S7 Western blot images/Figures S7. LNCaP-NC-shELOVL2.Tif]

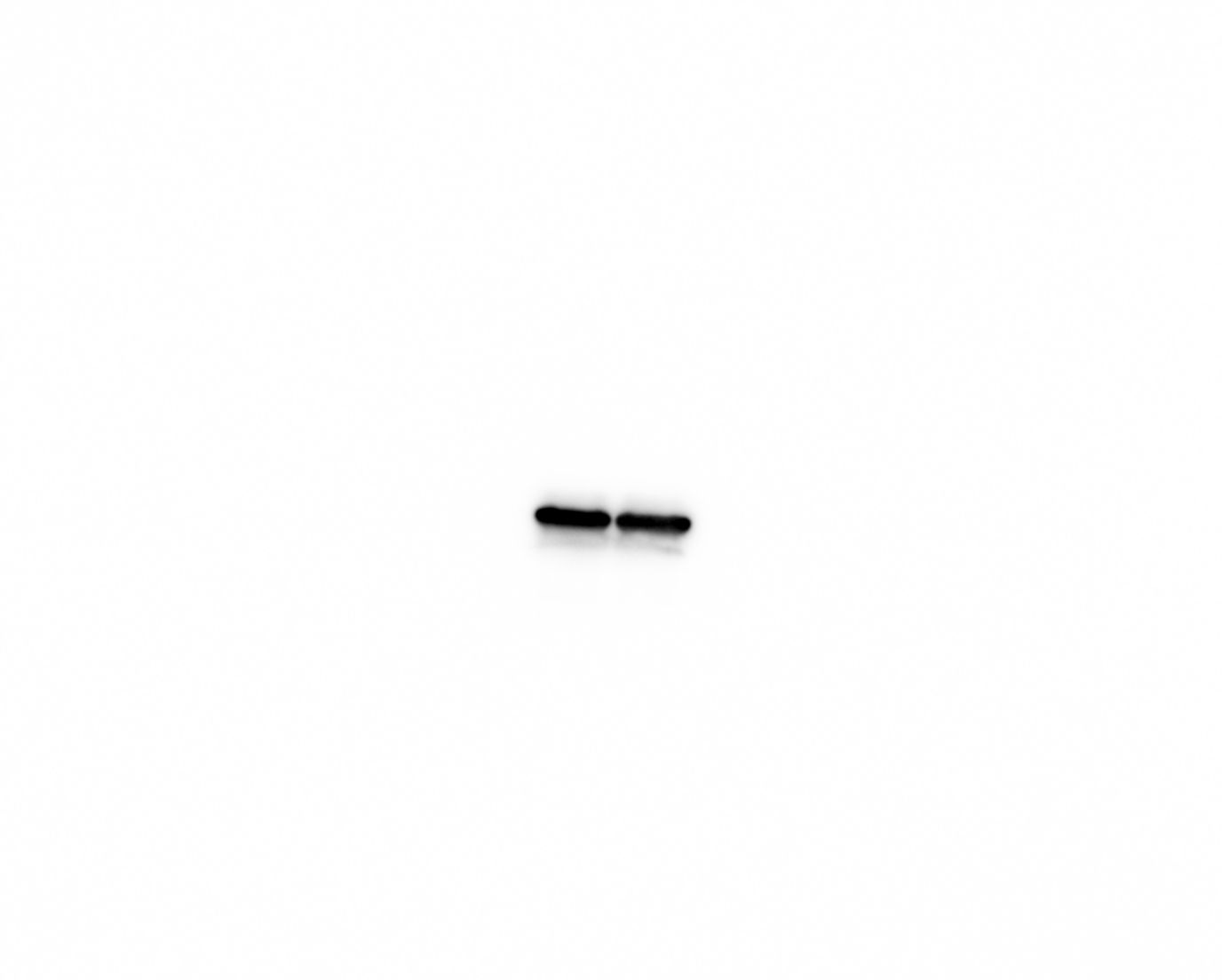

Supplement: Supplementary file 1 [file Supplementaryfile1.zip › Figure-Western blot images(revision-2025.05.22)/Figure S7 Western blot images/Figures S7. LNCaP-β-tubulin.Tif]

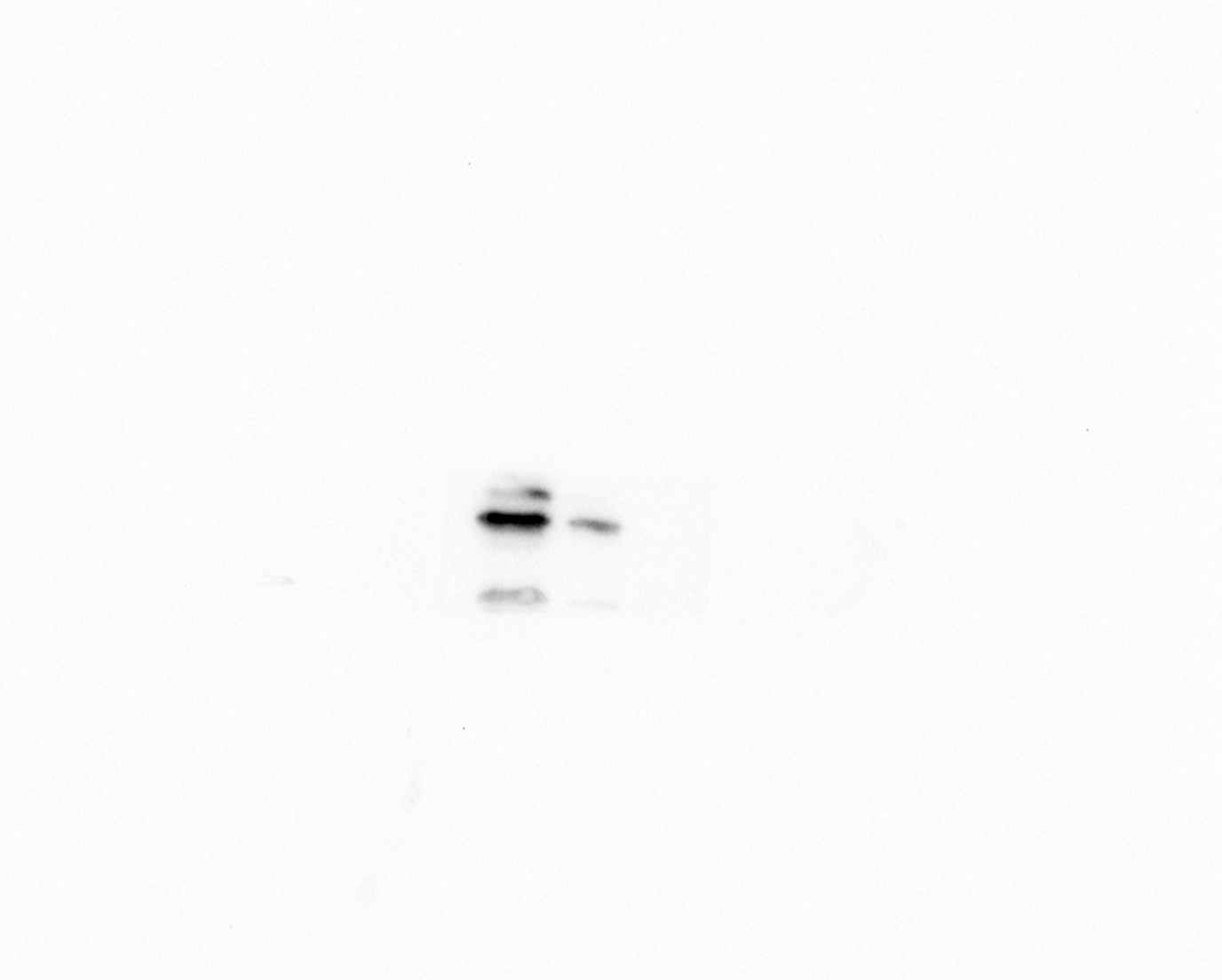

Supplement: Supplementary file 1 [file Supplementaryfile1.zip › Figure-Western blot images(revision-2025.05.22)/Figure S7 Western blot images/FigureS7.C4-2 -NC-shELOVL2.Tif]

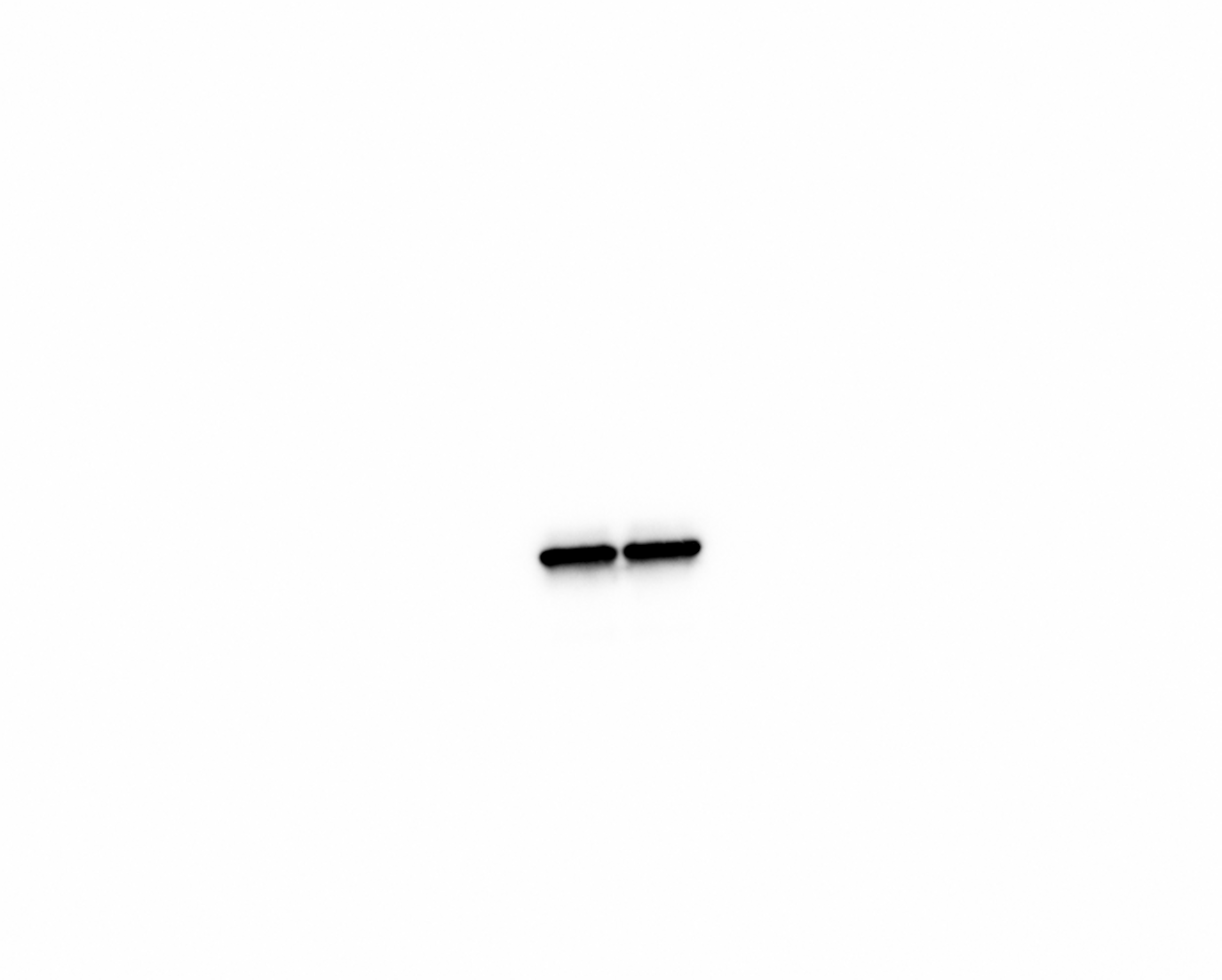

Supplement: Supplementary file 1 [file Supplementaryfile1.zip › Figure-Western blot images(revision-2025.05.22)/Figure S7 Western blot images/FigureS7.C4-2-β-tubulin.Tif]
