# Supplementary material for: ELOVL2 mediated stabilization of AR contributes to enzalutamide resistance in prostate cancer
Source: Front Cell Dev Biol. 2025 Jun 9;13:1598400. doi: 10.3389/fcell.2025.1598400 (PMC12183063; doi:10.3389/fcell.2025.1598400)
Supplement: Supplementary file 2 [file DataSheet1.docx]

Supplementary Material

**ELOVL2 Mediated Stabilization of AR Contributes to Enzalutamide Resistance in Prostate Cancer**

Jinpeng Cen^1, #^, Jiading Guo^2, #^, Xianzi Zeng^3, #^, Xianlu Song^4, #^, Shengdong Ge^1^, Mingkun Chen^5^, Qianyi Li^6^, Yuzhong Yu^1,^ *, Daojun Lv^7,^ *, Shanchao Zhao^1, 3,^ *

**Supplementary Table 1:Clinical Characteristics of Prostate Cancer Patients**

| **Patient** | **Age** | **Initial Diagnosis** | **Gleason Score** | **Source** |
| --- | --- | --- | --- | --- |
| **1** | 67 | Prostatic hyperplasia, elevated PSA under investigation | 3+3=6 | Post-prostatectomy pathology |
| **2** | 78 | Prostatic hyperplasia | 3+4=7 | Post-prostatectomy pathology |
| **3** | 81 | Prostate cancer | 4+4=8 | Post-prostatectomy pathology |
| **4** | 71 | Prostate cancer | 3+4=7 | Post-prostatectomy pathology |
| **5** | 77 | Prostatic hyperplasia | 4+5=9 | Prostate biopsy specimen |
| **6** | 69 | Elevated PSA under investigation | 3+4=7 | Prostate biopsy specimen |
| **7** | 84 | Bladder tumor, prostatic hyperplasia | 4+4=8 | Prostate biopsy specimen |
| **8** | 78 | Prostate cancer with widespread metastatic spread（ENZ-R） | 4+5=9 | Prostate biopsy specimen |
| **9** | 65 | Elevated PSA under investigation（ENZ-R） | 4+4=8 | Post-prostatectomy pathology |

**Supplementary Table 2: ELOVL2 siRNA Fragment Sequences**

| **Product No.** | **Product Name** | **Target Sequence** |
| --- | --- | --- |
| stB0011730A | genOFFTM st-h-ELOVL2_001 | GGTTCATGTTGGACTCTTA |
| stB0011730B | genOFFTM st-h-ELOVL2_002 | CAGTCATCTTATATGCTAA |
| stB0011730C | genOFFTM st-h-ELOVL2_003 | GCTACAACTTACAGTGTCA |

**Supplementary Table 3: Sequences of Primers Used for RT-qPCR**

| **Gene Name** | **Forward (F) Primer** | **Reverse (R) Primer** |
| --- | --- | --- |
| **ELOVL2** | GGAAGCTGACATCCGGGTAG | TCCAGTTCAAGACACACCACC |
| **GAPDH** | CTGGGCTACACTGAGCACC | AAGTGGTCGTTGAGGGCAATG |

**Supplementary FIGURE**

**
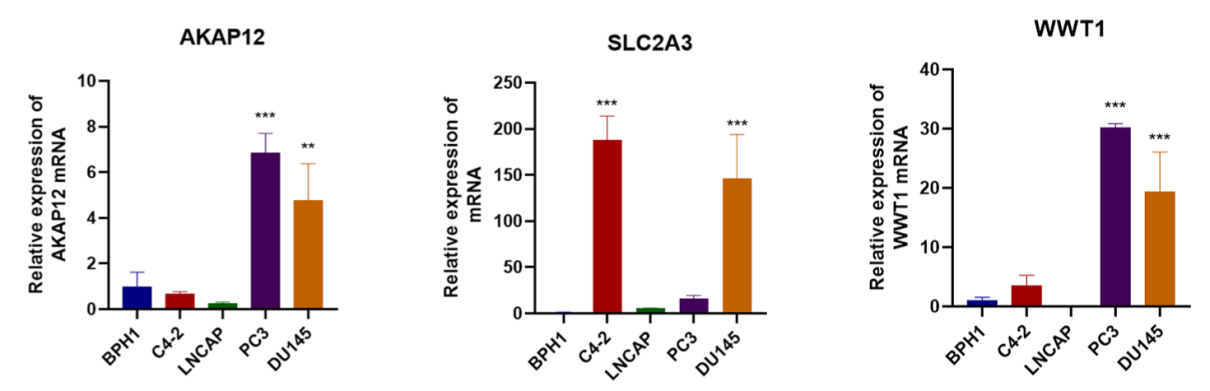
**

**FIGURE S1** qRT-PCR analysis of AKAP12, SLC2A3, and WWT1 expression in benign prostatic hyperplasia (BPH) tissues compared to prostate cancer (PCa) cell lines (LNCaP, C4-2, PC3, and DU145). Data are presented as mean ± SD; statistical significance is indicated as **P < 0.01, ***P < 0.001, and ****P < 0.0001.

**
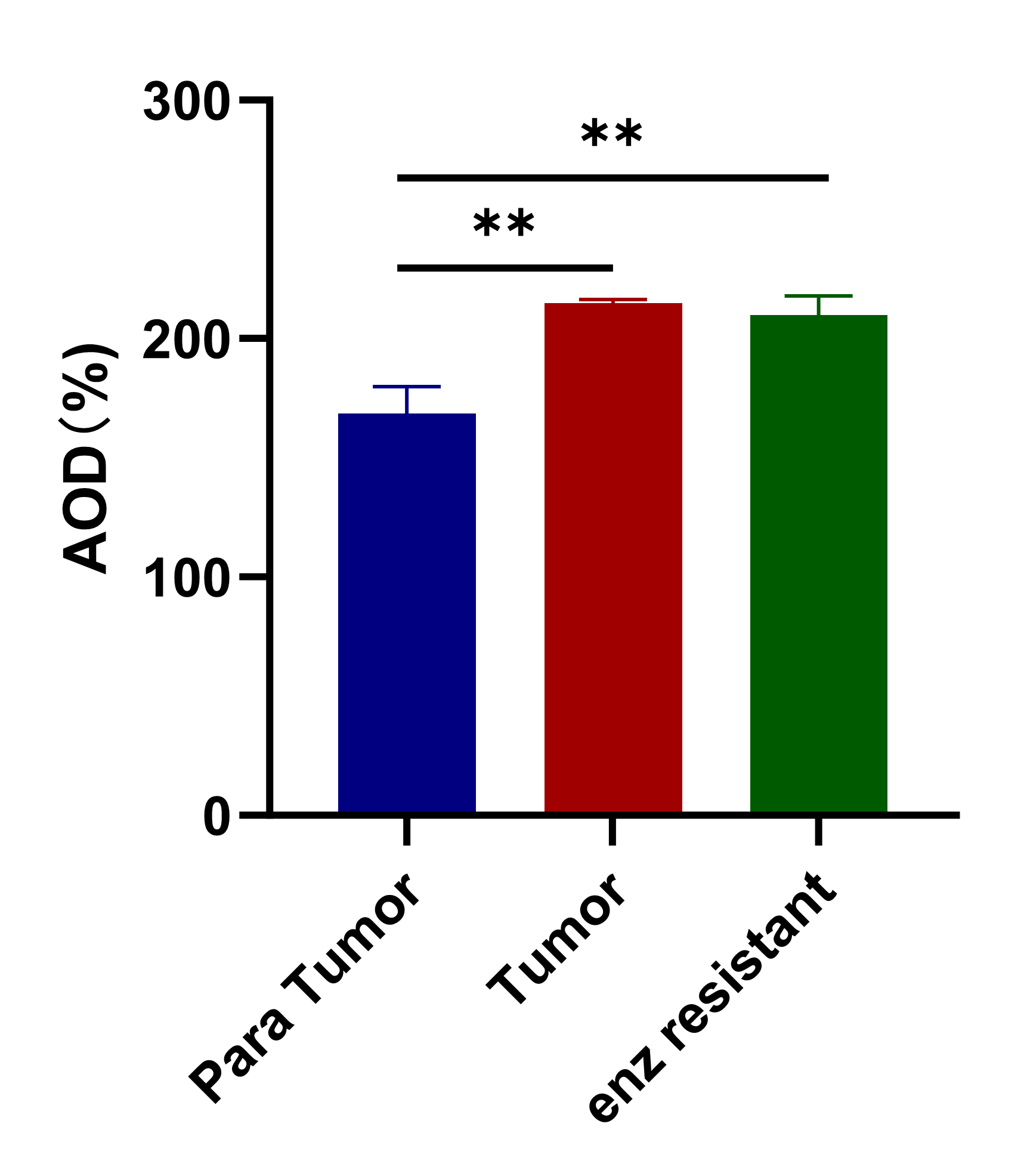
**

**FIGURE S2 Quantitative Analysis of Immunohistochemical Staining Intensity.**
Data represent the staining intensity of ELOVL2 in non-tumor tissues, primary PCa, and enzalutamide-resistant (ENZ-R) PCa tissues. Values are expressed as mean ± SD (n = 3 independent experiments). **P < 0.01 indicates statistically significant differences between groups (two-tailed Student’s t-test).

**
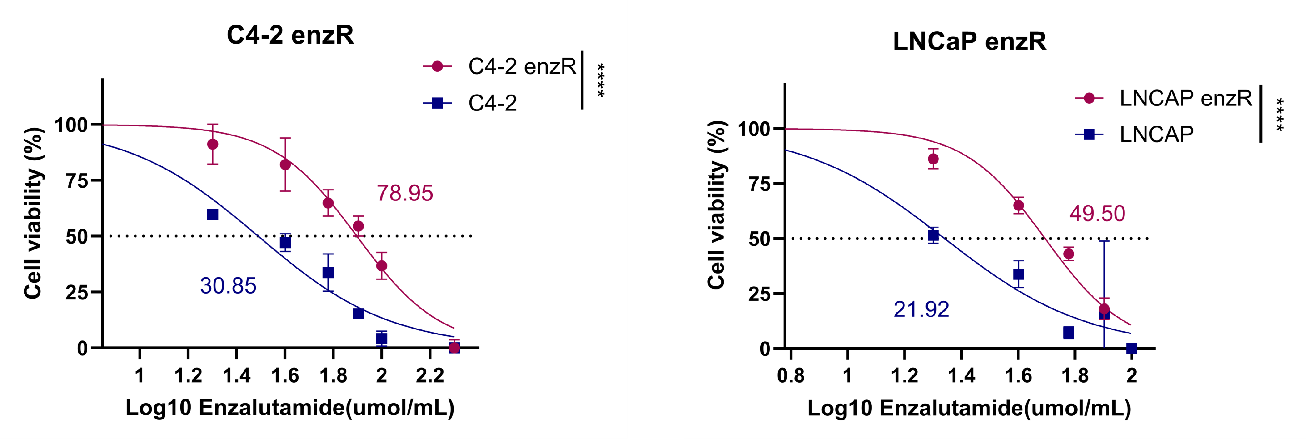
**

**FIGURE S3 Dose-response curves and IC₅₀ values of parental and enzalutamide-resistant (ENZ-R) cell lines after 7-day drug withdrawal.**

**Resistant cells maintained significantly higher IC₅₀ values (C4-2-enzR: 78.95 µM, 95% CI 73.88–84.35, ****P < 0.0001; LNCaP-enzR: 49.50 µM, 95% CI 46.12–52.79, ****P < 0.01) compared to parental cells (C4-2: 30.85 µM, 95% CI 27.05–34.59; LNCaP: 21.92 µM, 95% CI 15.17–27.52), confirming stable drug resistance**

**
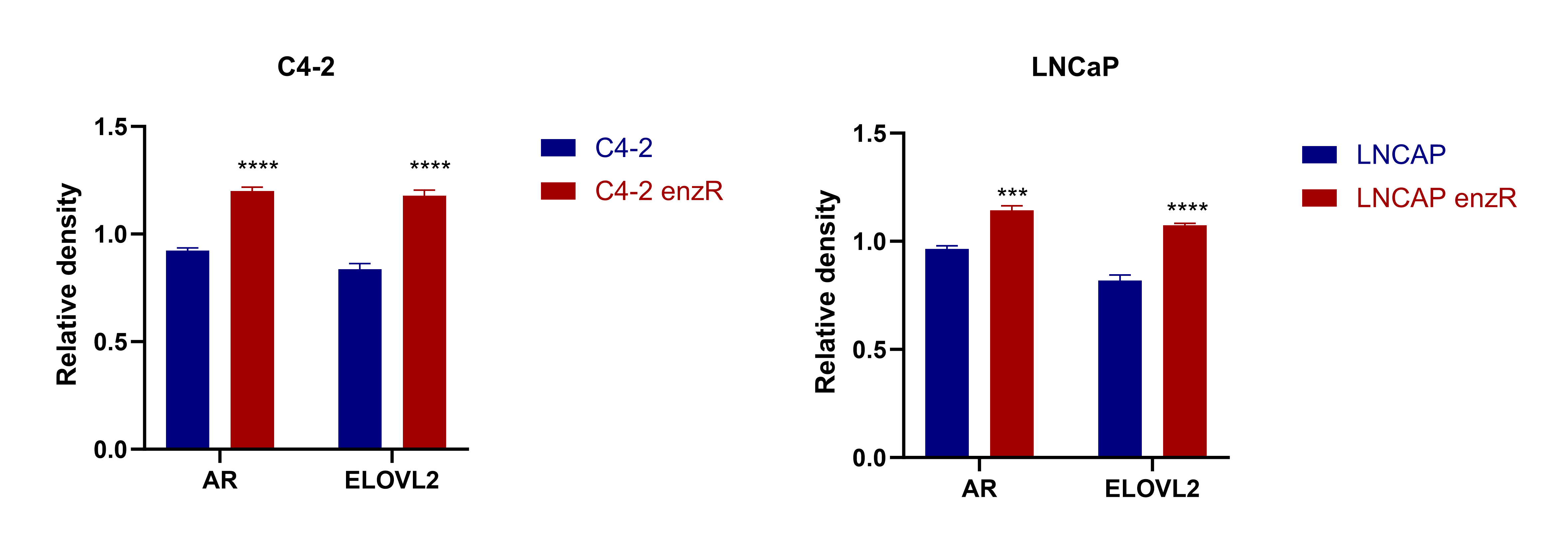
**

**FIGURE S4 Quantitative analysis of Western blot results shown in Figure 2E.** n=3, **Data are presented as mean ± SD. Statistical significance was determined by Student's t-test (***P < 0.001, ****P < 0.0001)**

**
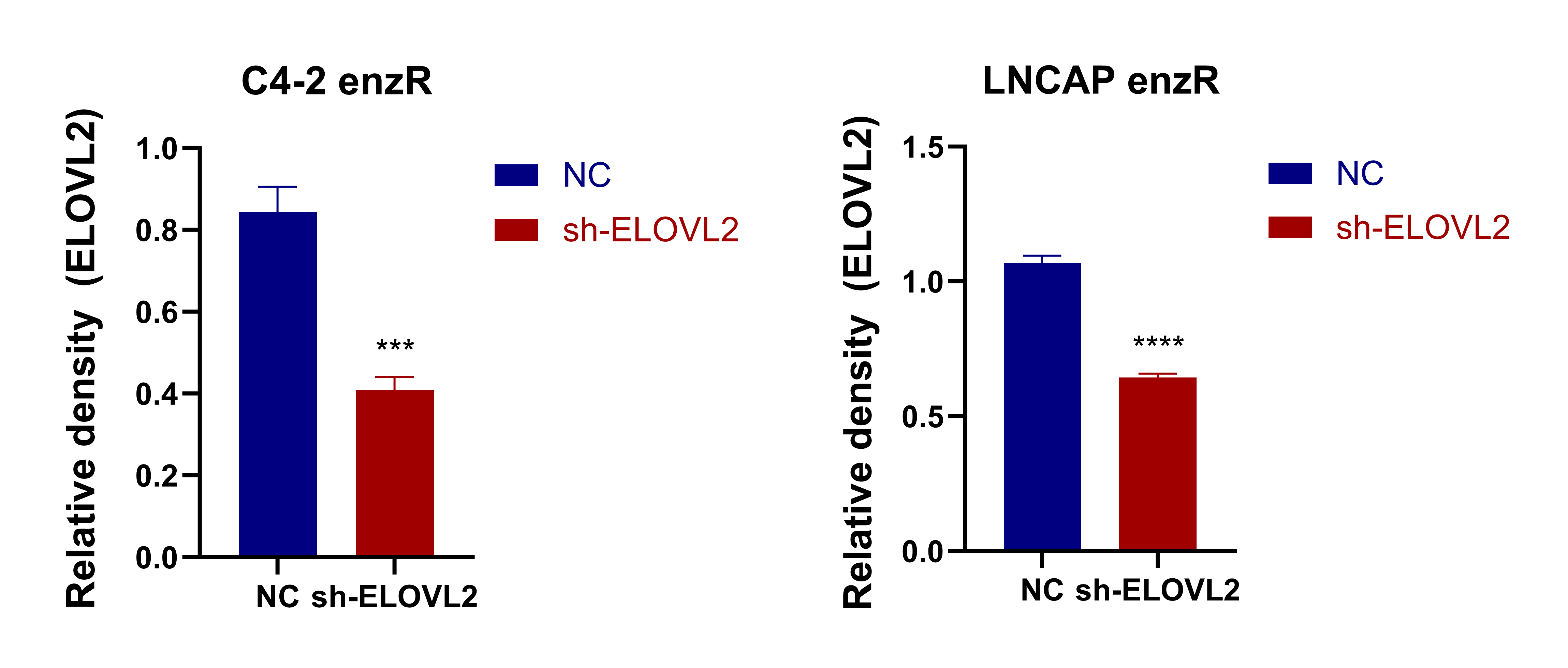
**

**FIGURE S5** Quantitative analysis of protein expression levels from Western blot in Figure 3B, n=3, Data represent mean ± SD. Statistical significance was assessed by two-tailed Student's t-test (***P < 0.001, ***P < 0.0001 ).

**
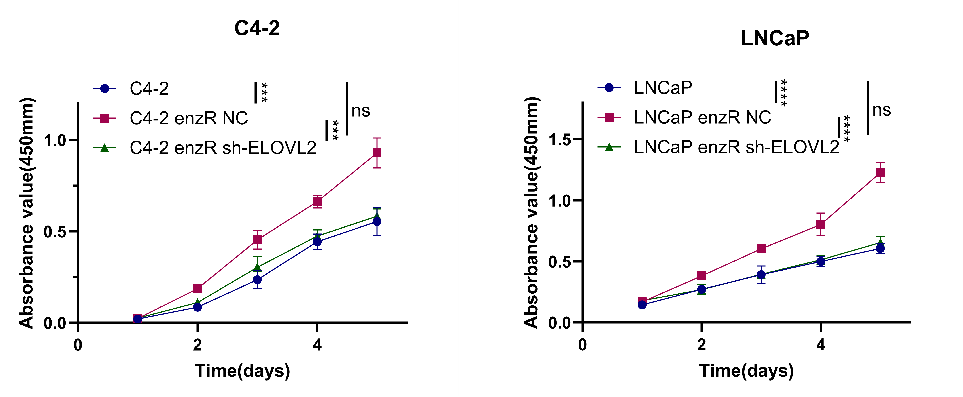
**

**FIGURE S6** Cell proliferation assessed by CCK-8 assay in C4-2, C4-2-enzR, and LNCaP-enzR cells following ELOVL2 knockdown. Data represent mean ± SD (n=5), ns: not significant ***p < 0.001.

**
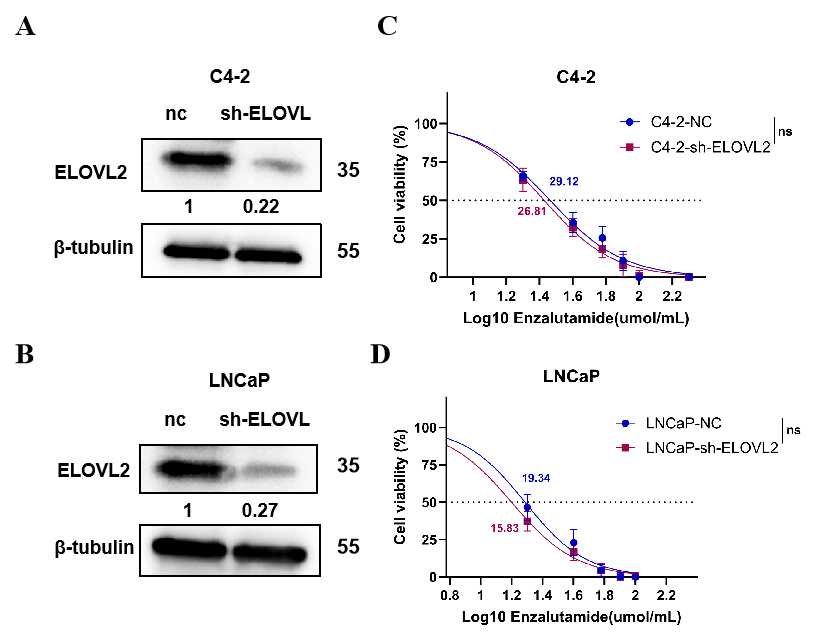
**

**FIGURE S7** (A, B) Western blot analysis of ELOVL2 protein levels in ELOVL2-knockdown C4-2 and LNCaP cells compared with control cells. (C, D) Dose-response curves and calculated half-maximal inhibitory concentration (IC50) values for enzalutamide in wild-type cell lines. No significant differences were observed in IC50 values between ELOVL2-knockdown and control cells for either C4-2 (26.81 µM, 95% CI: 24.55-29.05 vs 29.12 µM, 95% CI: 26.54-31.71; P > 0.05) or LNCaP (15.83 µM, 95% CI: 13.54-17.71 vs 19.34 µM, 95% CI: 16.65-21.69; P > 0.05)


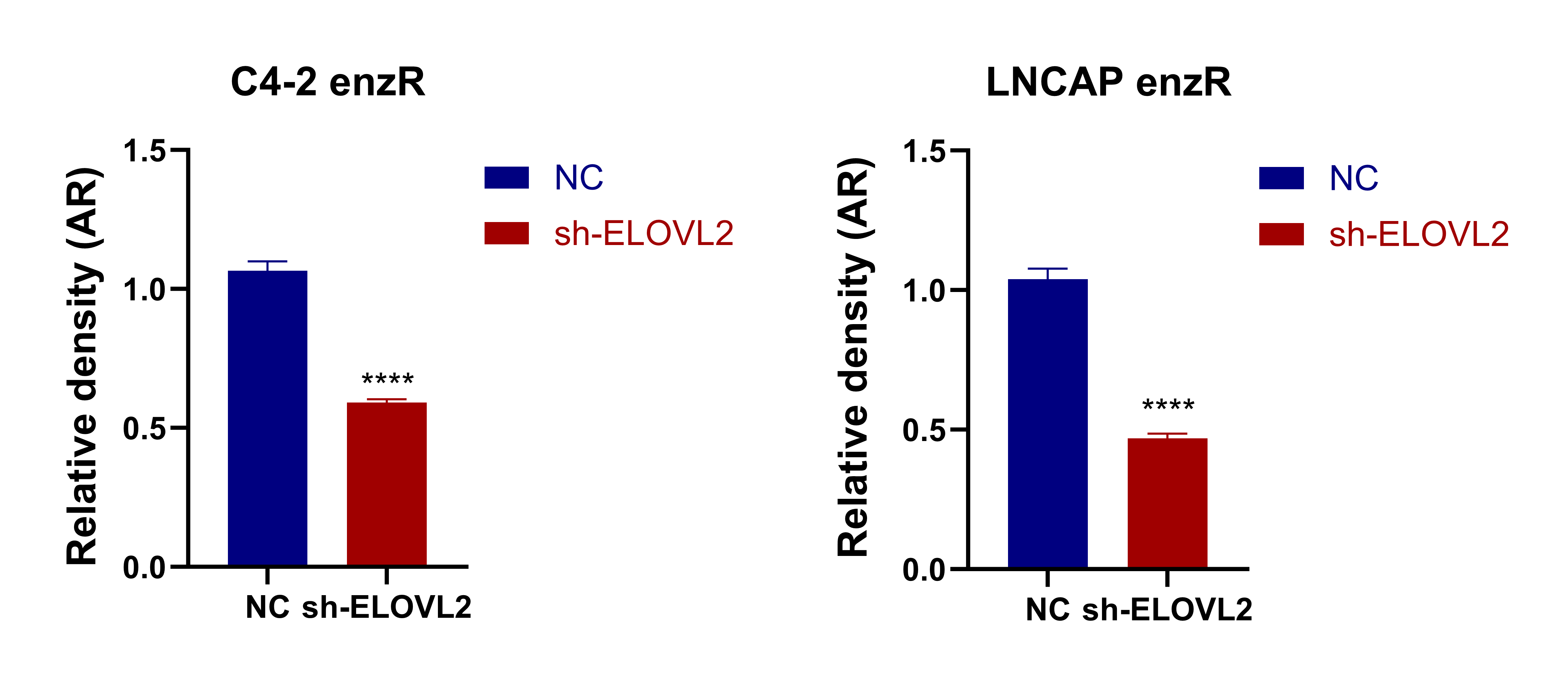


**FIGURE S8** Quantitative analysis of protein expression levels from Western blot in Figure 4B, n=3, Data represent mean ± SD. Statistical significance was assessed by two-tailed Student's t-test ( ****P < 0.0001 ).
